# Automated Label‐Free Assay for Viral Detection and Inhibitor Screening via Biomembrane‐Functionalized Microelectrode Arrays

**DOI:** 10.1002/adma.202501985

**Published:** 2025-08-14

**Authors:** Zixuan Lu, Jeremy Treiber, Konstantinos Kallitsis, Ekaterina Selivanovitch, Alexandra Wheeler, Maria Lopez‐Cavestany, Zhongmou Chao, Sarah L Barron, Ju An Park, Darius Hoven, Anna Scheeder, Aimee Withers, Becky M. Hess, Clemens F. Kaminski, Alberto Salleo, Anna‐Maria Pappa, Susan Daniel, Róisín M Owens

**Affiliations:** ^1^ Department of Chemical Engineering and Biotechnology University of Cambridge Philippa Fawcett Drive Cambridge CB3 0AS UK; ^2^ Department of Materials Science and Engineering Stanford University Stanford CA 94305 USA; ^3^ Robert F. Smith School of Chemical and Biomolecular Engineering Cornell University Ithaca NY 14853 USA; ^4^ Department of Biomedical Engineering Khalifa University of Science and Technology Abu Dhabi 127788 UAE; ^5^ Centre of Catalysis and Separations Khalifa University of Science and Technology Abu Dhabi 127788 UAE; ^6^ Pacific Northwest National Laboratory 902 Battelle Boulevard Richland WA 99 354 USA

**Keywords:** microelectrode array, microfluidics, PEDOT:PSS, SARS‐CoV‐2, SLB

## Abstract

Most virus infection assays have indirect readout such as virus number following entry (e.g., PCR, cell lysis). While effective, these technologies are labor‐intensive, require specialized environments (e.g., sterile or RNA‐free), and detect later‐stage viral events like lysis or cell death, lacking sensitivity to early fusion events. To address these limitations, we present biologically relevant 2D membrane materials, host‐cell‐derived supported lipid bilayers (hcd‐SLBs), integrated with organic microelectrode arrays (OMEAs) for detection of severe acute respiratory syndrome coronavirus 2 (SARS‐CoV‐2) fusion. By overexpressing angiotensin‐converting enzyme 2 (ACE2) receptors on the native membranes, the platform functions as a viral sensor capable of detecting virus pseudo particles (VPPs) through the late pathway. Additionally, hcd‐SLBs extracted from human lung epithelium expressing native ACE2 detect fusion events through the early pathway. The platform's utility as a drug‐screening tool is demonstrated by testing antibodies targeting either the ACE2 on the host membrane or the viral spike (S) proteins. To enhance the throughput, microfluidics are integrated for automation and OMEAs are incorporated within each channel, miniaturizing the testing units. This system supports high‐throughput data generation, automation, and scalability, providing an efficient platform for viral fusion detection that advances the study of pathogen‐host interactions and accelerates antiviral drug discovery.

## Graphical abstract

A bioelectronic sensor integrating 2D cell membranes with organic microelectrode arrays enables detection of SARS‐CoV‐2 fusion pathways. Natively‐expressed viral receptors promote monitoring of viral entry and screening of antibody‐based inhibitors. Integration with microfluidics further enhances throughput and usability, establishing a powerful platform for investigating viral fusogenicity and accelerating antiviral drug development.

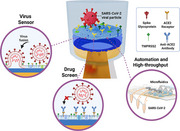

## 1 Introduction

Despite advances in vaccine development and deployment, the threat of future global pandemics looms large. A number of commercialized vaccines have been extremely effective, however there remains an urgent need for the development and selection of safe and effective therapeutics against viral infections to treat patients with severe symptoms or those who cannot take the vaccine due to underlying conditions.^[^ ^1^ ^]^

Host‐pathogen interactions can be studied using plasma membrane or endosomal membrane models as an alternative to whole‐cell assays. Since SARS‐CoV‐2 fusion occurs on host cell membranes, supported lipid bilayers (SLBs) derived from the host cell plasma membranes bearing the active receptors, represent a more simplified, but still physiologically relevant system to study fusion events without the logistics associated with the use of live cells.^[^ ^2^ ^]^ These host‐cell‐derived (hcd) SLBs, a new class of biological 2D materials, preserve membrane protein functionalities and maintain integrability to existing electronic platforms.

The hcd‐SLBs form via self‐assembly from blebs onto solid planar surfaces while maintaining the complexity of the “mother cell” membrane, including protein function, and orientation as well as membrane mobility and fluidity,^[^ ^3^, ^4^ ^]^ allowing for their interrogation using surface sensitive methods.^[^ ^5^ ^]^ Previous works used SLBs to study virus fusion, such as influenza,^[^ ^6^ ^]^ sindbis,^[^ ^7^ ^]^ and CoVs,^[^ ^2^ ^]^ and observed fusion events using total internal reflection fluorescence (TIRF) microscopy. The subsequent formation of these “native cell membranes” on electroactive surfaces, opened up a new direction where membrane events and their interactions with the environment could be electrically probed in a direct, lable‐free, and quantifiable manner.^[^ ^8^, ^9^, ^10^ ^]^ Using native SLBs interfaced with conducting polymer‐based (such as, Poly(3.4‐ethylenedioxythiophene) polystyrene sulfonate (PEDOT:PSS)) devices, we previously demonstrated electrical detection of influenza H3N2 VPP, strain X‐31, fusing with membranes derived from African green monkey (Vero E6) cells and baby hamster kidney (BHK) cells.^[^ ^11^ ^]^ These cell lines expressed sialic acid receptors, onto which the hemagglutinin (HA) proteins of the influenza virus are known to bind and initiate fusion.^[^ ^11^ ^]^ Further, we demonstrated mouse hepatitis (MHV)‐A59 VPP fusion on 17‐Cl1 mouse cell membranes.^[^ ^12^ ^]^ The principle for virus detection is postulated to be due to the incorporation of the viral membrane into the SLB upon fusion, resulting in an increase in membrane impedance.^[^ ^10^ ^]^

This approach was also recently used by us^[^ ^13^ ^]^ to demonstrate that the two biological entry pathways of coronavirus could be re‐created on this bioelectronic impedance platform and the resulting electrical signatures used to characterize unique fusion activity, or fusogenicity, of SARS‐CoV‐2 virus strains. In this work, SLBs were derived from Vero E6 cells overexpressing Transmembrane protease/serine subfamily member 2 (TMPRSS2) (representing the early pathway of viral entry) and compared with unmodified Vero E6 cells (representing the late pathway). The early pathway fusion was immediately detected electrically since the ACE2 and TMPRSS2^[^ ^14^, ^15^, ^16^ ^]^ were added in situ, whereas the late pathway fusion was sensed by recreating the endosomal compartment conditions with the addition of cathepsin L (protease)^[^ ^17^ ^]^ within an acidic environment.^[^ ^18^ ^]^ The platform could clearly distinguish the fusogenicity of SARS‐CoV‐2 Wuhan‐Hu‐1 (WH1) VPPs from Omicron variants, as well as differentiate the fusogencities between two Omicron subvariants, Omicron BA.1 and Omicron BA.4, aligning with known infectivity trends of these variants. These VPPs modified with viral binding proteins faithfully mimic native viruses and serve as an ideal model for fusogenicity research.^[^ ^19^, ^20^ ^]^

**Figure 1:**
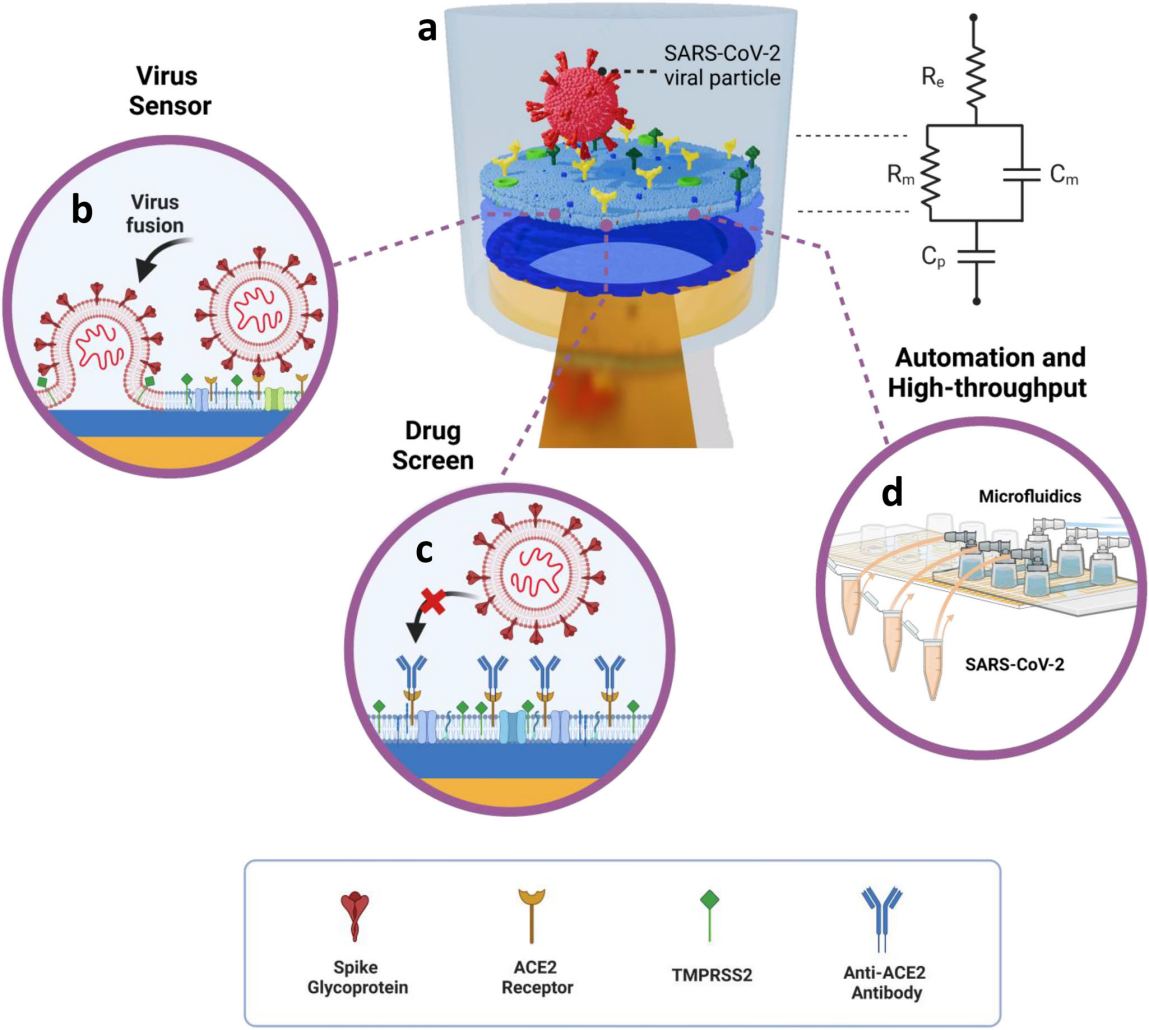
The three main application aspects of the SARS‐CoV‐2 functional assay on the biomembrane‐on‐chip system: a), virus particle attachment on native hcd‐SLB on a PEDOT:PSS microelectrode (left). The model circuit (right) represents the electrochemical properties of the system (R_e_, R_m_, C_m_, and C_p_ stands for electrolyte resistance, membrane resistance, membrane capacitance, and PEDOT:PSS capacitance, respectively). b), A sensor for detecting SARS‐CoV‐2 VPP fusion; c), A screening platform to study drug‐induced viral inhibition. d), An automated setup for high‐throughput/content viral infection search.

Pivoting from the previous work, we have now expanded on the potential of this platform for VPP detection and screening of viral inhibitors. Here, we first validated our previous results, reporting on two pathways of fusion activities of SARS‐CoV‐2 VPPs,^[^ ^13^ ^]^ but this time using HEK293‐ACE2 (human embryonic kidney cell SLBs with overexpressed ACE2 receptors for the late pathway) and Calu‐3 derived SLBs (for the early pathway). The Calu‐3 human airway epithelial cell line naturally expresses the essential ACE2 receptor and TMPRSS2 protease, making the Calu‐3 derived SLBs a relevant human model for detecting fusion of SARS‐CoV‐2 ^[^ ^21^, ^22^, ^23^ ^]^ (**Figure** **1**a). Further, VPP inhibition assays were demonstrated using two separate modalities: 1. anti‐ACE2 antibodies to block the host‐cell membrane receptors and 2. anti‐spike (Anti‐S) antibodies to block the VPPs (Figure 1c). Lastly, we monitor viral fusion events with three types of SLBs (HEK293‐ACE2, Calu‐3, and synthetic SLBs) on the same device integrated with microfluidic channels (Figure 1d). The characteristic impedance responses increase with the levels of ACE2 expression on the corresponding cell membranes. By duplicating the sensing units of transparent electrode arrays integrated with fluidic channels, the resulting device not only enables high‐throughput sensing with automated flow but also supports in situ optical microscopy for bioevent observation. Although here demonstrated for SARS‐CoV‐2 VPP detection as an example, this platform has the potential of being a commercialized research tool for other viruses and anti‐viral drugs when the hcd‐SLBs and conditions are changed to match the particular pathogens.

## 2 Results

### 2.1 Characterization of Cell‐Derived Blebs, hcd‐SLBs, and Electrical Monitoring of Viral Fusion

**Figure 2:**
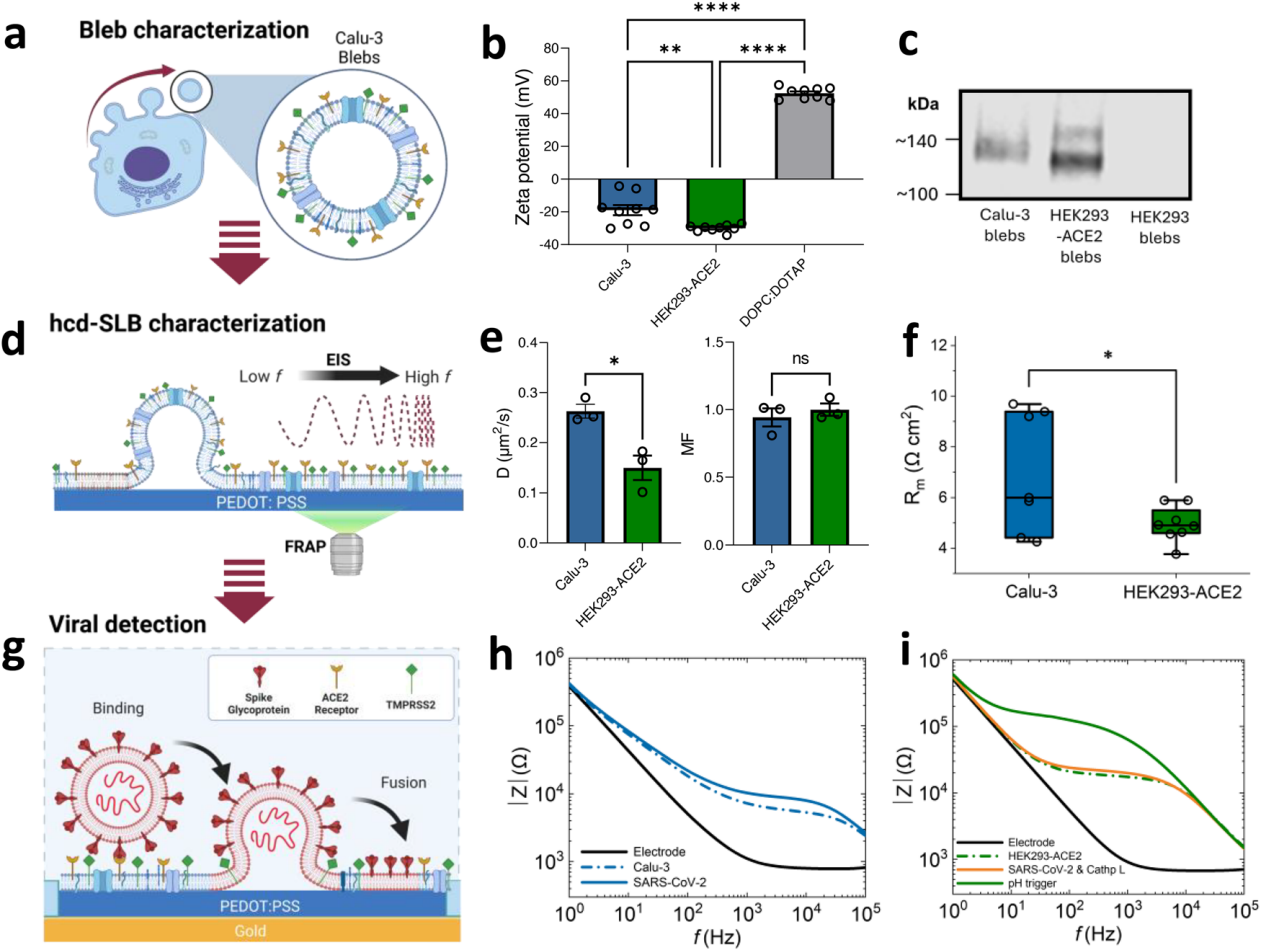
Integration and characterization of host cell membrane on PEDOT:PSS surfaces and SARS‐CoV‐2 VPP detection via biomembrane‐on‐chip. a–c), Characterization of blebs: (a), Schematics of mammalian cell (e.g., Calu‐3 cells) membrane vesicle extraction (blebbing). (b), Zeta surface charge of Calu‐3 blebs, HEK293‐ACE2 blebs, and DOPC:DOTAP liposomes are determined to be −18.9 ± 3.1 mV, −30.0 ± 0.71 mV, and 52.4 ± 1.2 mV, respectively (n = 3. Data points are technical repeats. The data points for each condition are tested for normal distribution and One‐Way ANOVA. P = 0.0014 for Calu‐3 and HEK293‐ACE2 blebs. *p* <0.0001 is both for the comparison between Calu‐3 and DOPC:DOTAP and the comparison between HEK293‐ACE2 and DOPC:DOTAP. Data are shown as mean ± s.e.m.). (c), Western blot showing both Calu‐3 and HEK293‐ACE2 blebs have expression of ACE2 receptors, indicated by the bands at ≈130 kDa (full blot in Figure S1, Supporting Information), whereas HEK293 cells show no detectable expression. d–f) optical and electrochemical characterization of Calu‐3 and HEK293‐ACE2 SLBs: d), schematic of reconstitution of biomembrane onto a PEDOT:PSS surface via the vesicle fusion method (sequentially adding fusogenic liposomes and polyethylene glycol to fuse native blebs). (e), Comparison of diffusivity (D) and mobile fraction (MF) values of Calu‐3 and HEK293‐ACE2 SLBs extracted from FRAP measurements with octadecyl rhodamine B chloride 18 (R18) stain (n = 3. The data points for each condition are tested by unpaired t‐test. p = 0.0159 for D comparison. p = 0.516 for MF comparison. Data are shown as mean ± s.e.m.). (f), Comparison of R_m_ of Calu‐3 and HEK293‐ACE2 SLBs. The mean R_m_ values extracted from Calu‐3 (6.97 ± 0.90 Ω cm^2^) and HEK293‐ACE2 (4.96 ± 0.25 Ω cm^2^) EIS measurements (n ≥ 3. Median values are represented by the lines within the box of each condition. Conditions passed normality tests and test with an unpaired t‐test and p = 0.0397). g–i), EIS characteristics for SARS‐CoV‐2 VPP fusion: (g), schematic showing the VPP binding and fusion conditions on Calu‐3 SLB (early pathway). Representative EIS measurements (Bode plots) of SARS‐CoV‐2 VPP fusion on Calu‐3 and HEK293‐ACE2 SLBs formed on opaque PEDOT:PSS electrodes: (h) fusion on Calu‐3; (i) fusion on HEK293‐ACE2 with addition of cathepsin L (orange curve) at low pH environment (solid green curve). (For all the biostatistics results: ns, *p* >0.05, ^*^, *p* ≤ 0.05; ^**^, *p* ≤ 0.01; ^***^, *p* ≤ 0.001; ^****^, *p* ≤ 0.0001).

First, the formation of membrane vesicles from Calu‐3 cells and HEK293‐ACE2 cells is chemically induced and extracted using a previously reported method (**Figure** **2**a).^[^ ^24^ ^]^ Native hcd‐SLBs are formed by fusion of the cell‐derived membrane vesicles/blebs (Figure 2d),^[^ ^8^, ^9^ ^]^ triggered by sequencially incorporating fusogenic compounds.^[^ ^10^ ^]^ This “sequential method” results in native Calu‐3 and HEK293‐ACE2 SLBs forming on PEDOT:PSS‐coated thin film surfaces. Nanoparticle tracking analysis (NTA) reveals bleb sizes ranging from 50–300 nm (Figure S2a,c, Supporting Information). The zeta surface charge of Calu‐3 and HEK293‐ACE2 blebs are determined to be −18.9 ± 3.08 and −29.9 ± 0.75 mV, respectively (Figure 2b), in agreement with the fact that cell surfaces are generally negatively charged.^[^ ^25^ ^]^ The difference in surface charge of the blebs could associate with a combined charge effect of various native cell‐membrane components (e.g., lipid or transmembrane proteins). Western blot analysis confirms the presence of the ACE2 receptor migration at ≈130 kDa in both cell lines (Figure 2c).^[^ ^26^ ^]^ HEK293‐ACE2 blebs, which overexpress ACE2, show a more intense signal than Calu‐3 blebs. Additionally, the human ACE2 receptor has a net negative charge in a pH 7.4 environment,^[^ ^27^ ^]^ likely contributing to the more negative zeta potential of HEK293‐ACE2 blebs.

Fluorescence recovery after photobleaching (FRAP) is used to assess the recovery of the fluorescence signal induced by lateral diffusion of unbleached lipids into a bleached spot,^[^ ^28^ ^]^ hence informing on mobility and diffusion properties of the Calu‐3 and HEK293‐ACE2 membranes (Figure S2b,d, Supporting Information). The diffusivity (D) and the mobile fraction (MF) were determined to be 0.26 ± 0.01 µm^2^ s^−1^ and 0.94 ± 0.12 for typical Calu‐3 SLBs, respectively, and for HEK293‐ACE2 SLB, the D and MF are 0.15 ± 0.02 µm^2^ s^−1^ and 0.99 ± 0.05, respectively (Figure 2e), which are in line with literature reported values on PEDOT:PSS surfaces.^[^ ^11^, ^12^ ^]^ HEK293‐ACE2 SLBs exhibit lower fluidity (i.e., lower D) compared to the Calu‐3 SLBs, which is likely due to the more negative charge on their membrane compared to Calu‐3, resulting in stronger electrostatic repulsion to the excess of negatively charged PSS, found in commercially available PEDOT:PSS.^[^ ^10^, ^29^ ^]^ Probing the formation of cell‐derived SLBs and native membrane‐protein associated bio‐events on PEDOT:PSS coated microelectrodes via EIS allows us to sense and extract the barrier properties (e.g., membrane resistance (R_m_)) of an SLB with an equivalent circuit model (Figure 1a).^[^ ^10^ ^]^ In agreement with the D values, the HEK293‐ACE2 SLBs (4.96 ± 0.25 Ω cm^2^) formed on PEDOT:PSS thin film surfaces have a slightly lower average R_m_ compared to Calu‐3 SLBs (6.97 ± 0.90 Ω cm^2^) (Figure 2f).

**Figure 3:**
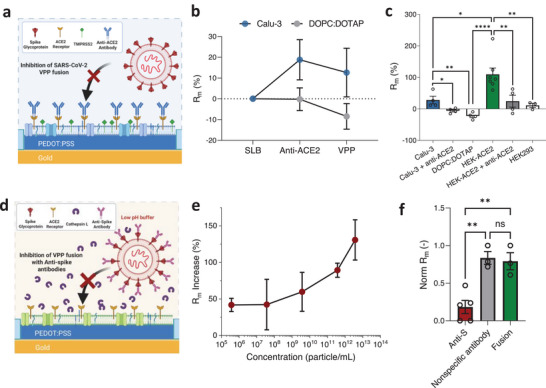
SARS‐CoV‐2 inhibition assay with antibodies: a) Schematic of inhibition of SARS‐CoV‐2 fusion by anti‐ACE2 antibodies binding onto the ACE2 receptors on the Calu‐3 membrane. b) Percentage change in R_m_ of Calu‐3 and DOPC:DOTAP SLBs after incubation with anti‐ACE2 antibodies for 1.5 h (at room temperature) followed by addition of VPPs (≈10^11^ particle mL^−1^). c) R_m_ percentage change after addition of SARS‐CoV‐2 VPPs (≈10^11^ particle mL^−1^) to different SLB types and conditions (1.9–2.3 µg mL^−1^ anti‐ACE2 antibodies were added for each antibody treated condition. n ≥3. The data points for each condition are tested for One‐Way ANOVA; p = 0.0337 is between Calu‐3 and Calu‐3 + anti‐ACE2; p = 0.0037 is between Calu‐3 and DOPC:DOTAP; p = 0.0095 is between HEK293‐ACE2 and HEK293‐ACE2 + anti‐ACE2; p = 0.0061 is between HEK293‐ACE2 and HEK293; p = 0.0139 is between Calu‐3 and HEK293‐ACE2. *p* <0.0001 is between DOPC:DOTAP and HEK293‐ACE2.) d) Schematic demonstrating inhibition of SARS‐CoV‐2 VPP fusion by neutralising the spike proteins with anti‐spike antibody. e) The percentage membrane resistance increase induced by VPP fusion at different concentrations. f) Normalised Rm (unitless) comparison between HEK293‐ACE2 SLBs tested with VPPs (3.67 × 10^5^ particle mL^−1^) incubated with 10 µg mL^−1^ anti‐S antibody (red bar, labeled as anti‐S), with 10 µg mL^−1^ anti‐GFAP as a nonspecific antibody (grey bar, labeled as nonspecific antibody), and pristine VPPs (~3.67 × 10^5^ particle mL^−1^ green bar, labeled as fusion) (n ≥ 3. The data points for each condition are tested for One‐Way ANOVA; p = 0.0022 is between anti‐S and fusion conditions; p = 0.0014 is between Anti‐S and nonspecific antibody conditions. n.s. represents not significant, ^*^, *p* ≤ 0.05; ^**^, *p* ≤ 0.01; ^***^, *p* ≤ 0.001; ^****^, *p* ≤ 0.0001). (Data are presented as mean ± s.e.m.).

Following the same principle, EIS is a quantitative method to observe VPP fusion with hcd‐SLBs. Generally, SARS‐CoV‐2 enters the host cell membrane via its spike (S) proteins in two key steps (Figure S3a, Supporting Information): 1. binding onto the host cell receptor and 2. triggering viral membrane fusion with the host cell membrane. As Calu‐3 cells express both ACE2 receptors and TMPRSS2 proteases on their cell membrane, it is a suitable model to study the early pathway of SARS‐CoV‐2 entry processes (Figure 2g; Figure S3a, Supporting Information). When the SARS‐CoV‐2 VPPs are added to the Calu‐3 SLBs, the viral entry step immediately begins^[^ ^30^ ^]^ (see Bode and Nyquist plots in Figure 2h; Figure S3b, Supporting Information). The fusion of the VPPs caused a 28.2 ± 12.0% increase of the R_m_ compared to the baseline of Calu‐3 SLBs (blue bar in **Figure** **3**c). To the best of our knowledge, this is the first demonstration of the detection of viral‐particle fusion with receptors at native‐expression levels via the human hcd‐SLB‐on‐chip system. Furthermore, the late pathway viral‐entry processes (Figure S3a, Supporting Information) were mimicked by adding VPP, cathepsin L (protease), and acidic buffer (pH trigger buffer with pH 4.5) sequentially on to HEK293‐ACE2 SLBs to fulfil the late pathway conditions.^[^ ^11^, ^30^ ^]^ A 109.5 ± 20.2% increase in R_m_ with respect to the pristine HEK293‐ACE2 SLBs was caused by viral fusion (green bar in Figure 3c). The HEK293‐ACE2 SLB model serves as both a positive control and a model for the late pathway of viral entry. The total response in HEK293‐ACE2 SLBs shows a 3.9× increase in Rm compared to Calu‐3, due to the higher ACE2 expression in HEK293 cells. These results are consistent with a previous study,^[^ ^13^ ^]^ though variations may arise from differences in protein expression levels and SLB formation methods (see method session). Negative controls using HEK293 and lipid‐only SLBs showed negligible Rm changes, highlighting the necessity of ACE2 receptors for viral fusion.

### 2.2 Screening Inhibitors of SARS‐CoV‐2 Fusion through ACE2 Receptor and Spike‐Protein Blocking

The development of platforms to screen antibodies or anti‐viral drugs is crucial especially when such viruses represent a threat to human life. The sensitivity and reproducibility are powerful features of the OMEA‐based sensor for screening drug molecules. Herein, we carry out a SARS‐CoV‐2 VPP inhibition assay with commercially available anti‐ACE2 and anti‐S antibodies, as a pseudo drug to show the feasibility of our biomembrane electronic assay as a fast and sensitive screening platform.

Since binding between the S‐protein and ACE2 receptor is the initial step in virus fusion, we performed a viral inhibition assay with anti‐ACE2 antibodies (Figure 3a) blocking subsequent VPP attachment to ACE2. When the anti‐ACE2 antibodies are incubated with Calu‐3 SLBs, there is an 18.8 ± 9.7% increase in R_m_, likely attributable to the binding of the anti‐ACE2 antibodies with the ACE2 receptors (Figure 3b; Figure S4ai,aii, Supporting Information). No changes were noted on control (lipid‐only, DOPC:DOTAP) SLBs. Anti‐ACE2 antibodies likely provide extra barriers to the ionic current, resulting in the R_m_ extracted from EIS measurements being higher.^[^ ^8^ ^]^ Addition of VPPs to the antibody‐treated SLB did not induce any further increase in R_m_ (Figure 3b; Figure S4a, Supporting Information). Instead, we noticed a negligible decrease (less than 6%), which was also observed in the control SLB (DOPC:DOTAP) (Figure 3b; Figure S4bi,bii, Supporting Information).^[^ ^31^ ^]^ These results indicate that inhibition of VPP fusion can be detected using these devices. The same trend was observed with HEK293‐ACE2 SLBs: the anti‐ACE2‐antibody‐blocked HEK293‐ACE2 SLBs demonstrated a significantly lower R_m_ increase compared to the unblocked HEK293‐ACE2 SLBs (Figure 3c) when VPPs were added. Additionally, the blocked HEK293‐ACE2 SLBs demonstrated similar behavior to HEK293 SLBs (lacking ACE2 receptors), highlighting that blockage by the anti‐ACE2 antibodies prevents the binding and fusion events of the VPP (Figure 3c; Figure S5, Supporting Information) as detected by this platform.

To detect inhibition of VPP fusion by blocking the S‐protein, HEK293‐ACE2 was chosen as the testbed since it exhibited a more pronounced EIS response to VPP fusion (see impedance increase in Figure 3c). This allows us to discriminate impedance changes induced by inhibition of VPP entry, from the control (non‐treated VPPs). However, when high VPP concentrations (e.g., ≈10^9^ and ≈10^7^ particle mL^−1^) were used, the effects of significant inhibition cannot be detected even with a relatively high concentration of anti‐S antibody (10 µg mL^−1^) (see Figure S9, Supporting Information). According to previous neutralisation study of influenza viruses, to detect a significant neutralisation effect of the virus, the anti‐S antibody number should exceed the number of S proteins on VPPs in the sample.^[^ ^32^ ^]^ Significant inhibition of VPP entry can however be observed when lower concentration of VPPs are incubated with an anti‐S antibody (10 µg mL^−1^). Hence, we need to accurately know the concentration of VPPs and dilute accordingly for the inhibition test. The traditional NTA‐based methods for determining nanoparticle concentration can introduce significant systemic error.^[^ ^33^ ^]^ To achieve more accurate VPP concentration measurements and establish a reliable detection range for this platform, we implemented a strategy inspired by cell‐counting with hemocytometers (see method section and Equation S1, Supporting Information) to accurately determine the concentration of VPP using structured illumination microscopy (SIM).^[^ ^34^ ^]^ As a super‐resolution imaging technique with a resolution ≈100 nm, SIM is capable of visualizing VPPs ranging from several tenths to a few hundredths of nanometres in size (Figure S7, Supporting Information).^[^ ^35^ ^]^ This particle quantification method is compatible with various fluorescence microscopy systems and is not limited to SIM. However, employing a super‐resolution system (such as SIM) enhances the reliability of quantifying higher particle concentrations (Figure S8, Supporting Information). Therefore, we suggest that particle concentrations can be assessed more accurately using a super‐resolution microscope. With this direct‐counting method, the concentration of the testing batch of VPPs was determined to be 3.67 × 10^13^ particle mL^−1^, which is 1000× fold higher than the NTA result (Figure S7, Supporting Information). A series of pristine VPP concentrations was tested on HEK293‐ACE2 SLBs with EIS. As Figure 3e shows, the impedance change caused by fusion has a clear dose‐dependent trend. The lower the VPP concentration, the lower the increase in the R_m_ induced by VPP fusion. This trend is likely highly correlated to the amount of viral material introduced to the SLB after the fusion process. A VPP sample with ≈3.67 × 10^5^ particle mL^−1^ was still seen to trigger a 41.8 ± 9.0% increase in R_m_, using our highly sensitive sensing platform. The data in Figure 3e show a modest change in R_m_ increase at VPP concentrations between 10⁵ and 10⁷ particles mL^−1^, followed by a sharp rise at higher concentrations. This trend may reflect the complex and potentially cooperative nature of virus–host membrane binding kinetics,^[^ ^36^ ^]^ which involve dynamic processes.^[^ ^37^ ^]^

Compared to the VPPs incubated with anti‐S antibodies, there was more than a threefold increase in R_m_ with pristine VPPs (Figure 3f; Figure S9, Supporting Information). When the VPPs were incubated with non‐specific antibodies (anti‐GFAP antibody is applied as a control in Figure 3f) at the same concentration (10 µg mL^−1^) as the anti‐S antibodies, the increased value of R_m_ was similar to fusion induced by the VPPs without any antibody added. Further, since the antibodies are all dissolved in PBS, the HEK293‐ACE2 SLBs were washed and incubated (10 min) with PBS to check whether this process has an impact on the SLB. As Figure S11 (Supporting Information) shows, no significant difference in R_m_ was observed after this process. Therefore, this inhibition effect is specifically associated with the blocking of S‐proteins by anti‐S antibodies.

### 2.3 Automated Viral Tests with Multi‐Microfluidic Channel System

**Figure 4:**
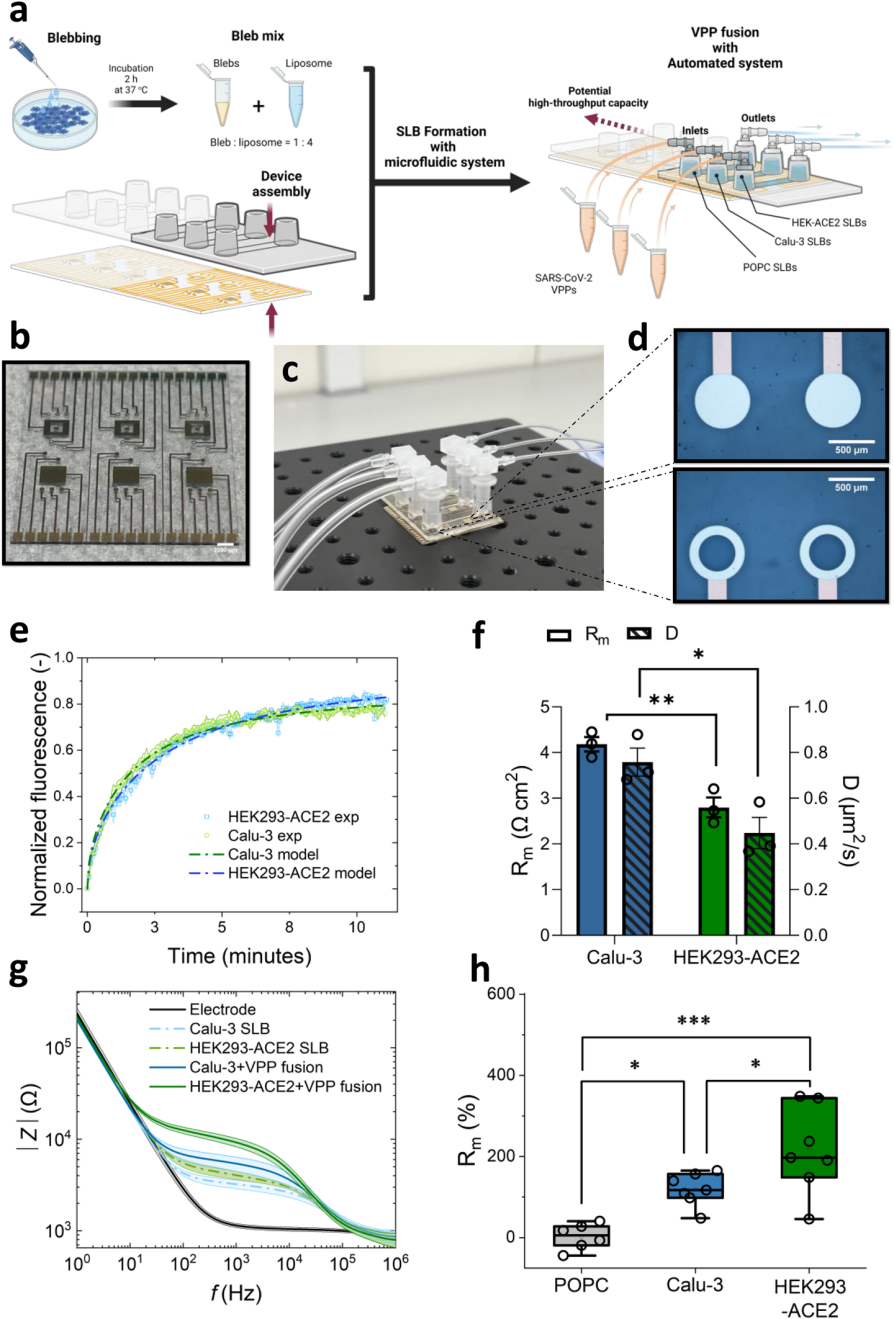
VPP fusion tests on multiple types of SLBs with microfluidic‐channel delivery: a), a schematic demonstrating bleb mix preparation, assembly of ibidi^®^ microfluidic channels with the microelectrode array, formation of SLBs, and testing VPPs on three types of SLBs. b), the microelectrode array chip with solid and ring electrodes arranged based on the ibidi^®^ microfluidic channels. c) The assembled device with microfluidics and tubing connected. d) The micrographs of solid PEDOT:PSS electrodes (top) and ring PEDOT:PSS electrodes (bottom). e) The FRAP curves of Calu‐3 and HEK293‐ACE2 SLBs through ring electrodes in channels adjacent to each other within the same chip (data are shown as mean ± s.e.m, n = 3 electrodes. Doted curves are the average of experimental values, the error bars are presented as shaded areas, mean ± s.e.m. The dashed curves are average values from model fitting). f) The correlation of diffusivity (D) and R_m_ on the same ring electrode array (n = 3. The comparison of D and R_m_ are tested by a separated unpaired *t*‐test. p = 0.275 for D; p = 0.0069 for R_m_. Data are shown as mean ± s.e.m.). g) Curves of EIS responses of VPPs fusion on Calu‐3 and HEK‐ACE2 SLBs (Data are shown as mean ± s.d., n ≥ 4 for technical repeats with two biological repeats). h) percentage change of R_m_ after VPPs fused on three types of SLBs (POPC (1‐oleoyl‐2‐palmitoyl‐sn‐glycero‐3‐phosphocholine) is neutrally charged and chosen as a control, which is a more biologically‐relevant and lipid‐only model of mammalian cell membrane.^[^ ^39^ ^]^ n ≥ 6. The data points for each condition are tested for normal distribution and One‐Way ANOVA. P = 0.0212 is between POPC and Calu‐3; p = 0.0001 is between POPC and HEK293‐ACE2; p = 0.0481 is between Calu‐3 and HEK293‐ACE2. ^*^, *p* ≤ 0.05; ^**^, *p* ≤ 0.01; ^***^, *p* ≤ 0.001; ^****^, *p* ≤ 0.0001).

Furthermore, we demonstrated the potential of our platform for scalability and automation by integrating our microelectrode arrays with commercial ibidi^®^ microfluidic channels (**Figure** **4**a–d). This integrated multi‐electrode‐microfluidic system enabled a more controlled testing environment and increases both measurement throughput and sample size, leading to more accurate, reproducible, and potentially commercializable assays for viral fusion and antiviral screening. The microfluidic‐channel slides were easily integrated with our microfabricated OMEAs by attaching the adhesive layer to the chip and maintaining pristine surfaces of the arrays, ensuring robust bonding while eliminating the risk of fluid leakage and accidental viral exposure. However, adapting the “sequential method” of SLB formation with a microfluidic system proved challenging. Therefore, we applied a “premixing protocol” (see Experimental Section: VPP fusion test on three types of SLBs with microfluidic system), which increased the chance of SLB formation with a microfluidic‐channel setup (Figure 4a; Video S1, Supporting Information).

We also designed and microfabricated OMEAs with a circular‐shape (solid electrode) (Figure 4d top) and ring‐shaped electrodes (Figure 4d bottom), arranged at opposite ends of the chip. Both electrode types had the same PEDOT:PSS area (600 µm diameter), but the ring electrodes featured a transparent window for optical monitoring of SLBs. No significant difference in impedance or SLB formation preferences were observed between the two electrode types. The optical transparency of ring electrodes allowed us to correlate the optical (FRAP) and the electrical (EIS) properties of an SLB on the same electrode. Following the same trend presented in Figure 2e, the D and R_m_ appeared to correlate with bleb surface charges (Figure 4e,f). HEK293‐ACE2 SLBs, with an overexpression of ACE2 receptors and a more negatively charged surface, showed lower values of D compared to Calu‐3 SLBs, indicating less favourable diffusion of lipids in HEK293‐ACE2 SLBs (Figure 4e). The more negatively charged surface on HEK293‐ACE2 may result in a reduction of effective packing of lipid and cell membrane components on the negatively charged electrode surface (due to an excess amount of PSS), resulting in a lower barrier effect of HEK293‐ACE2 SLBs. Therefore, this was shown as a slightly lower R_m_ and D compared to Calu‐3 SLBs (Figure 4f).

Next, we conducted simultaneous characterization of SARS‐CoV‐2 VPPs on three types of SLBs on a chip with automated delivery of reagents via the microfluidic setup to demonstrate how a high throughput assay would be set up and executed. Compared to the synthetic SLB, where no VPP fusion was detected (≈2.6% increase), fusion effects on both Calu‐3 and HEK293‐ACE2 SLBs were detected by EIS, with R_m_ changes related to ACE2 receptor expression (Figure 4g,h). The R_m_ percentage changes of Calu‐3 and HEK293‐ACE2 SLBs were 119.1 ± 15.1% and 216.0 ± 40.5%, respectively, following the trend with the tests performed on static systems (Figure 2f). The increased sensitivity was likely due to the controlled and more targeted reagent delivery (with constant flow rates) via the microfluidic setup. Additionally, we utilised large PEDOT:PSS counter electrodes (Figure 4b; Figure S12, Supporting Information) confining the electrochemical circuit for testing SLBs within the microfluidic channel. The distance between the working and counter electrode affects the overall impedance values, so the working (ring and solid) electrodes were designed to have a similar distance to the shared counter electrode (the two counter electrodes are shared for the electrodes in proximity). In Figure S13 (Supporting Information), SLBs recorded by PEDOT:PSS electrodes with the co‐planar counter electrode (two‐electrode configuration) had a similar absolute value of membrane resistance as the “three‐electrode” configuration, but showed slight shifting of the spectra. This means that the two‐electrode configuration is valid and accurate for measuring membrane impedance. Importantly, this co‐planar counter electrode setup enhances portability, for point‐of‐care applications by avoiding the traditional bulky “three‐electrode” configurations.^[^ ^38^ ^]^

## 3 Conclusion

This work presents a biomembrane integrated electronic platform to facilitate the detection and screening of anti‐virals for SARS‐CoV‐2 viral entry. Herein, we first characterized the blebs and membranes from two different cell lines, one naturally expressing the ACE2 receptor (Calu‐3) as well as one engineered to overexpress it (HEK293‐ACE2). Second, we sensed both early and late pathways of SARS‐CoV‐2 fusion, which matches our previously reported results, specifically, we detected fusion with the cell membranes having natively‐expressed receptors. Third, we detected inhibition of viral entry with antibodies that specifically bind ACE2 receptors or S‐proteins. These results demonstrate proof of concept that this platform can be used for the detection of receptor‐specific drugs/antibodies, which could even be applied for screening the affinity and effectiveness of antibodies. Lastly, the microfluidic‐on‐chip setup further improves the throughput, ease‐of‐use, and sensitivity for sensing viral fusion.

In terms of the electronic readout, the fusion response of early pathway detection on Calu‐3 SLBs is relatively lower than the responses on HEK293‐ACE2 SLBs as the late‐pathway counterpart. However, the Calu‐3 SLB model, with its native ACE2 expression, offers a realistic and clinically relevant approach for COVID‐19‐related research^[^ ^30^ ^]^ and drug development. In contrast, the HEK293‐ACE2 SLBs offer a sensitive detection model, capable of identifying viral presence at concentrations of 10⁵ particles mL^−1^ and above.^[^ ^40^ ^]^ As a sensitive detector, HEK293‐ACE2 SLBs integrated with OMEAs could also be developed into a time‐efficient and point‐of‐care tool complementary to well‐established infectious diagnostics, such as polymerase chain reaction (PCR). This platform also could be used to rapidly screen existing or newly‐developed therapeutics against SARS‐CoV‐2 variants. However, it is important to acknowledge that this method would not be appropriate for assessing antivirals that interfere with cellular metabolites, underscoring the value of the live cell system as a complementary approach to address this limitation.

Moreover, the SIM‐based VPP counting method represents a pioneering approach to accurately quantify nanoparticle concentrations from fluorescent signals. Unlike many commercial methods prone to systematic errors, this super‐resolution microscopy technique holds significant promise as a foundational experimental tool, which warrants further investigation for advancing nanoparticle and nano‐medicine research. Lastly, the microfluidic system integrated with OMEAs provides an automated and user‐friendly setup for high‐throughput viral testing by streamlining the process from SLB formation to VPP detection. Testing three types of hcd‐SLBs demonstrated the system's scalability for viral and drug testing by incorporating additional units. Its versatility allows integration of various human‐cell‐line‐derived membranes, creating a body‐on‐chip platform for comprehensive analysis of pathogen or drug effects on the human body. This biomembrane‐on‐chip system shows significant potential for rapid drug screening and high‐throughput diagnostics, offering a transformative tool against virus‐induced diseases.

## 4 Experimental Section

### Cell Culture

Human lung epithelial cells, Calu‐3 cells (ATCC, Cat no. HTB‐55, RRID: CVCL 0609, Passage number 24–34) were cultured with RPMI 1640 media (Gibco) supplemented with 10% FBS (Merck), 1% penicillin/streptomycin (10000 U mL^−1^; TFS), and 1% GlutaMax (TFS). Cells were maintained at 37 °C and 5% CO_2_. Media was replaced every 2–3 days, and cells were passaged with 0.25% trypsin‐EDTA (TFS) when 80–90% confluent.

Human embryonic kidney 293 cells overexpressed with ACE2 receptor (HEK293‐ACE2 cells, transfected and shipped from the lab at University of Cornell) were cultured in 25 cm^2^ flasks with Dulbecco's modified Eagle medium (DMEM; Thermo Fisher Scientific, TFS) with the addition of 10% foetal bovine serum (Merck), 50 U mL^−1^ penicillin and 50 µg mL^−1^ streptomycin (TFS), 1% (v/v) GlutaMax (TFS), and 50 µg mL^−1^ gentamicin (TFS). Cells were cultured in an incubator (at 37 °C, with 5% CO_2_, and in humidity) until 80–90% confluent before passaging.

### Preparation of Plasma Cell Membrane Vesicles (Blebbing)

Calu‐3 and HEK293‐ACE2 cells were seeded in culture dishes (10 cm diameter, Corning) or T25 flasks (DMEM; Thermo Fisher Scientific, TFS) and grown until desired confluency at 37 °C and 5% CO_2_. Cells were then washed with GPMV buffer (2 mm CaCl_2_, 10 mm HEPES, 150 mm NaCl at pH 7.4) and then incubated with 4 mL (2 mL for T25 flask) of GPMV buffer supplemented with 25 mm formaldehyde (FA, Sigma‐Aldrich, F8775) and 2 mm dithiothreitol (DTT, Sigma‐Aldrich, 43816) to induce the formation of blebs, for 2 h at 37 °C. The solution containing the blebs was collected and placed on ice for 15 min to separate cell debris from the blebs, which were subsequently collected from the supernatant.

### Fluorescence Recovery after Photobleaching Measurements (FRAP)

FRAP experiments were performed on an inverted Zeiss LSM800 confocal microscope (Zeiss Germany) with a 10× objective. To label the membrane, 1 µL of 0.36 mm R18 (ThermoFisher Scientific, Cat. no. O246) fluorophore was added to 200 µL of bleb or liposome solution in a soft sonication bath for 15 min. The excess fluorophore was removed by centrifuge (3000 rpm, 3 min) using a G25 spin column (Cytiva, Cat. no. 27532501). Starting from the labeled blebs, supported lipid bilayers were assembled as described. A 150 mW 561 nm optically pumped semiconductor laser (Coherent, Inc.) was used to photobleach a 30 µm diameter spot in the supported lipid bilayer and its fluorescence intensity recovery was monitored up to around 20 min. The fluorescence intensity change over time was fitted using a Bessel function, following the method of Soumpasis.^[^ ^28^ ^]^ The diffusion coefficient was calculated with the following equation:

(1) $$D=w^{2}/4t_{1/2}$$

where *w* is the radius of the photobleached spot and *t*_1/2_ is the time required to achieve half of the maximum recovery intensity.

### Fabrication of Microelectrode Array

The devices were fabricated with “parylene C lift‐off method” and “PEDOT:PSS etching method,” and a detailed method is aviable in Supporting Information.

### Formation of Native Supported Lipid Bilayers (SLBs)

100 µL of blebs were added to PEDOT: PSS‐based electrodes after 1 min of O_2_ plasma (Diener electronic) at 25 W and 0.8 mbar. The blebs were incubated for 10–15 min and then rinsed with PBS 3 times. All‐lipid liposomes (DOPC/DOTAP 4 mg mL^−1^ in PBS) were added and incubated for 20 min and then rinsed with PBS (3 times). A poly(ethylene glycol) (Thermo Fisher, BP23‐1) solution in PBS (PEG8k, 30% w/v) was then added to the well and incubated for another 20 min. Lastly, the SLBs were washed with PBS 5 times before measurements.

### Electrochemical Impedance Spectroscopy with Potentiostat

An Autolab PGSTAT204 potentiostat equipped with a frequency response analyser was used to record impedance spectra in the frequency range between 1 Hz–100 KHz. Commercial Ag/AgCl and a platinum mesh were used as reference and counter electrodes, respectively. The micro‐fabricated PEDOT:PSS coated Au electrodes were used as the working electrode. An AC voltage of 0.01 V and a DC voltage of 0 V versus OCP (open circuit potential) were applied. All measurements were taken in ≈200 µL PBS retained on the chip by a glass well.

For the EIS measurement monitoring SLBs formed on the microfluidic‐on‐chip system, an Autolab PGSTAT302N potentiostat was used to record impedance spectra in the frequency range between 1–10^6^ Hz. The micro‐fabricated electrode PEDOT:PSS electrodes were used as working electrodes, and the PEDOT:PSS gate within the same plane were used as the counter electrode (shorted with the reference connector). An AC voltage of 0.01 V and a DC voltage of 0 V versus OCP (open circuit potential) were applied.

### Preparation of SARS‐CoV‐2 Virus Pseudotyped Particles

Human embryonic kidney cells HEK293 cells were seeded on 6‐well plates with 2 mL of C480 DMEM solution per well. The cell density typically reached ≈50% confluence prior to proceeding to the next step. Transfection was performed with three plasmids encoding for the different proteins required to form pseudotyped particles: the envelope glycoprotein, MLV gag and pol proteins, and luciferase reporter. The total amount of DNA per well was 1 µg with 300 ng of gagpol, 400 ng of luciferase reporter, and 300 ng of the envelope protein. First the plasmids encoding for gagpol and luciferace were combined and incubated at room temperature for 5 min. For a 50 mL solution, 1.25 mLs of optimem and 1.4 mLs of polyethyleneimine (PEI) were added to a 50 mL falcon tube. The envelope proteins were added to the tube as well, appropriately scaling the amount to the 50 mL total volume. The envelope proteins were either SARS‐CoV2 Wuhan‐Hu‐1 spike protein, vesicular stomatitis virus (VSV, positive control) G glycoprotein, or a negative control that lacked any enveloped glycoproteins (ΔEnv, negative control) (Figure S6b, Supporting Information).^[^ ^13^ ^]^ The backbone proteins (gagpol) were then added to the same tube and incubated at room temperature for 20 min. F‐DMEM was added to a final volume of 50 mLs after the incubation. The C‐DMEM was aspirated from the HEK293 cells and washed with F‐DMEM prior to adding the transfection mixture. The F‐DMEM mixture was then added, where each well on the plate contained a final volume of 2 mLs, and incubated for 48 h at 37 °C. By the end of the incubation period, the cells typically changed color to orange, being careful not to over‐incubate (resulting in yellow color). The supernatant was collected from the wells and placed into 50 mL falcon tubes. These tubes were centrifuged for 7 min at 290 xg at 4 °C. Being careful not to disturb the bottom of the tubes, the supernatant was, once again, recovered and filtered through a 0.22 µm syringe filter. To ensure longevity of the samples, 1 mL aliquots were frozen and stored at −80 °C until needed for use.

Here, the presence of non‐VPP vesicles released from the plasma membrane was highlighted. A previous study by Lavado‐Garcia et al.^[^ ^41^ ^]^ estimated that non‐VPP vesicles comprise ≈30% of the total vesicle population after filtering. These vesicles are particularly challenging to isolate using current laboratory techniques.

### Determination of VPP Concentration with Structured Illumination Microscopy (SIM)

5 µL of 0.36 mm R18 was added to 50 µL of the original VPP sample and the mixture was placed in a soft sonication bath for 10 min. Excess R18 was removed by centrifuge (3000rpm, 3min) with a G25 spin column. Next, the VPP samples were serial diluted (Figure S7, Supporting Information), and 0.5 µL of the diluted sample was added on to a high precision cover slip, with a second cover slip being placed on top of the sample. VPPs were left to spread between the two cover slips for a period of 1 min, and the VPP containing cover slips placed on the SIM sample stage for imaging.

A 1.2 NA 60x water immersion lens (UPLSAPO 60XW, Olympus) was used to project structured illumination patterns onto the sample and the emitted fluorescence light was captured by an sCMOS camera (Orca‐flash 4.0, Hamamatsu). The wavelength of the excitation laser was 561 nm (OBIS 561, Coherent) for the R18 labeled VPPs. SIM reconstructions were carried using the open source FairSIM software package,^[^ ^42^ ^]^ following best practices for parameter selection.^[^ ^43^ ^]^ The microscopy experiments were performed in a temperature‐controlled room at 20 °C.

The SIM images of diluted VPP samples were in 8‐bit format and analysed via ImageJ. Particles were identified using the built‐in auto‐threshold function. The “watershed” algorithm was chosen to differentiate proximate particles. VPP counting was processed by selecting the function “Analyse Particles” with sizes ranging between 0.0079 and 0.05 µm^2^ (≈100–252 nm diameter, respectively) (Figure S7, Supporting Information).

The determination of the VPP sample spreading area between two cover slip was acquired by image process through ImageJ. The image was first adjusted to a black and white image (white area is the VPP spreading area) in Microsoft PowerPoint by selecting “Paint strokes” and “black and white: 50%.” Then, the image was imported to ImageJ to measure the white area by setting the scale from the length of the cover slip edge (22 mm).

### Fusion of VPP with Series Dilution

This process is tested on HEK293‐ACE2 SLBs. The original VPP sample was diluted with PBS into a series of concentrations: 10^12^, 10^11^, 10^9^, 10^7^, and 10^5^ particle mL^−1^. Each testing concentration was added to the SLBs after the baseline measurement of the HEK293‐ACE2 SLBs. Then, the step‐by‐step fusion process was the same as the discription in the additional experimental section in Supporting Information: Studies of two pathways of VPP entry.

### VPP Inhibition by ACE2 Antibody Binding on the HEK‐ACE2 SLB

Before the regular fusion process, the formed SLB (Calu‐3 or HEK293‐ACE2) were incubated for 1 h with an anti‐ACE2 antibody solution (1.9–2.3 µg mL^−1^ (1:100 diluted) in PBS. Abcam, ab108252.) at room temperature. The step‐by‐step fusion process were the same as the discription in the additional experimental section in Supporting Information: Studies of two pathways of VPP entry).

### VPP Inhibition by Anti‐Spike Antibody Blocking

This experiment was performed on HEK293‐ACE2 SLB incubated with 10 µg mL^−1^ anti‐SARS‐CoV2 spike glycoprotein S1 antibody (Abcam, ab273073). First, the VPP needed to be diluted to the desired concentration. Then, 10 µL anti‐SARS‐CoV2 spike glycoprotein S1 antibody (1 mg mL^−1^) was added into 990 µL diluted VPP with the desired concentration (the antibody is 10 µg mL^−1^), and this mixture was incubated for 1 h at 37 °C in a water bath. For the control experiment, GFAP monoclonal antibody (ThermoFisher Scientific, 13–0300) is diluted to 10 µg mL^−1^ in the VPP sample with the desired concentration. This mixture was incubated for 1 h at 37 °C in a water bath before testing. The incubated VPP sample was added to the HEK293‐ACE2 SLB for tests. The step‐by‐step fusion processes were the same as the discription in the additional experimental section in Supporting Information: Studies of two pathways of VPP entry).

### VPP Fusion Test on Three Types of SLBs with Microfluidic System

The formation of native SLBs from Calu‐3 and HEK293‐ACE2 on the OMEAs using a microfluidic system using the premixing method. First, the blebs were sonicated 5 min before being centrifuged the blebs (3000 rpm, 3 min) through the G25 spin columns. The blebs were then mixed with liposomes (POPC, Avanti Polar Lipids, 850457C) at a 1:4 ratio (60 µL blebs are added in 240 µL POPC) and sonicated for 10 min. The OMEA chips are soaked in 70% ethanol overnight before use. The ibidi microfluidic slides (sticky‐Slide VI 0.4, Cat. no. 80608) heated to 90 °C on a hot plate to soften the adhesive layer. PDMS (polydimethylsiloxane) wells were also attached to each array, and the OMEA chips were treated with O_2_ plasma (1–2 min, 25 W, 0.8 mbar) while the PDMS well were still in place. After the O_2_ plasma treatment, PDMS wells were quickly removed, and the ibidi microfluidic slides were pressed onto the chip.

The tubings were connected to the microfluidics via an elbow luer connector (ibidi, Cat. no. 10802). ≈200 µL of HEK293‐ACE2 mixture, Calu‐3 mixture, and POPC were delivered onto each OMEA at 500 µL min^−1^ flow rate with a syringe pump. After 30 min of incubation, 200 µL of the mixture were pumped in at with 500 µL min^−1^ flow rate to wash away exceeds blebs or liposomes. Next, 200 µL of 10wt.% PEG8k solution in PBS are flowed into each channel at 50 µL min^−1^ and incubated for 15 min. Each channel was then washed with PBS at a 500 µL min^−1^ flow rate before testing with VPPs.

Prior to the VPPs, the SLBs are incubated and stabilised within PBS for ≈30 min, and EIS baseline spectra are recorded. VPPs (100 µL min^−1^ flow rate) were then flowed into each channel and incubated for ≈15 min before EIS measurement. For the HEK293‐ACE2 SLBs, another set of EIS measurements was also recorded after incubated with 200 µL of pH4.5 buffer mixed with 1 µL 0.5 mg mL^−1^ cathepsin‐L/MEP protein (Abcam, ab81780). The electrode baselines were measured in PBS after incubating each OMEA with 70% ethanol overnight.

Additional experimental methods are available in the Supporting Information.

### Statistical Analysis

The pre‐processing of data (including normality test), sample sizes, statistical methods, and data presentation are detailed in the captions of each figure corresponding to the respective plots. Statistical analyses, including the normality test and one‐way ANOVA, were conducted using GraphPad Prism (graphpad.com).

## Conflict of Interest

The authors declare no conflict of interest.

## Author Contributions

Z.L., K.K., E.S., Z.C., S.D., R.M.O. conceptualized the whole project and the experiments. Z.L. designed and micro‐fabricated the OMEAs used in Figures 2, 3, and J.T. designed and micro‐fabricated the OMEAs used in Figure 4. Z.L. conducted the blebbing, zeta surface potential measurements. E.S. made the VPPs. A Wheeler contributed to the NTA for the blebs and VPPs. K.K. formed the first HEK293‐ACE2 SLBS and initiated the VPP fusion on HEK293‐ACE2 SLBs. Z.L. initiated the formation of Calu‐3 SLBs and VPP tests on Calu‐3 SLBs and contributed to the further work, including FRAP and EIS of all the types SLBs in this paper, VPP fusion tests, VPP inhibition experiments with anti‐ACE2 and anti‐S antibodies, and the VPP fusion experiments with microfluidic channel integrated OMEAs. J.A.P. and S.L.B. contributed to culturing Calu‐3 cells in the early stage of the project. Z.L. and A.W. contributed to culturing and maintaining the cells throughout the project. Z.L. and M.L.C. performed the western blots. Z.L. contributed to data plotting, data analysis, biostatistics, and arrangement of the figures, as well created the schematics for each figure. D.H. created the Blender 3D schematic in Figures 1a and 3D printed multichannel adapters for pumping fluid into microfluidic channels. A.S. helped with the determination of VPP concentration with SIM at C.F.K's lab. The first draft and last draft were written and checked by Z.L. and R.M.O., and edited by all the co‐authors. The project was supervised by R.M.O., S.D., A.M.P., A.S, and B.M.H.

## Supporting information



Supporting Information

Supplemental Video 1

## Data Availability

The data that support the finding of this study are openly available in Apollo (CAM) at, https://doi.org/10.17863/CAM.117824.
