# Supplementary material for: Automated Label‐Free Assay for Viral Detection and Inhibitor Screening via Biomembrane‐Functionalized Microelectrode Arrays
Source: Adv Mater. 2025 Aug 14;37(51):e01985. doi: 10.1002/adma.202501985 (PMC12721210; doi:10.1002/adma.202501985)
Supplement: Supplementary file 1 — Supporting Information [file ADMA-37-e01985-s001.docx]

Supporting information

**Automated Label-Free Assay for Viral Detection and Inhibitor Screening via Biomembrane-Functionalized Microelectrode Arrays**

*Zixuan Lu, Jeremy Treiber, Konstantinos Kallitisis, Ekaterina Selivanovitch, Alexandra Wheeler, Maria Lopez-Cavestany, Zhongmou Chao, Sarah L Barron, Ju An Park, Darius Hoven, Anna Scheeder, Aimee Withers, Becky M. Hess, Clemens F. Kaminski, Alberto Salleo, Anna-Maria Pappa, Susan Daniel, Róisín M Owens**

Z. Lu, K. Kallitisis, A. Wheeler, M. Lopez Cavestany, S. L. Barron, J. A. Park, D. Hoven, A. Scheeder, A. Withers, C. F. Kaminski, R. M. Owens

Department of Chemical Engineering and Biotechnology, University of Cambridge, Cambridge CB3 0AS, United Kingdom.

E-mail: [rmo37@cam.ac.uk](mailto:rmo37@cam.ac.uk)

J. Treiber, A. Salleo

Department of Materials Science and Engineering, Stanford University, Stanford, California 94305, United States.

E. Selivanovitch, Z. Chao, S. Daniel

Robert F. Smith School of Chemical and Biomolecular Engineering, Cornell University, Ithaca, New York 14853, United States.

A.M. Pappa

Department of Biomedical Engineering, Khalifa University of Science and Technology, 127788, Abu Dhabi, United Arab Emirates

Centre of Catalysis and Separations, Khalifa University of Science and Technology 127788, Abu Dhabi, United Arab Emirates.

B.H. Hess

Pacific Northwest National Laboratory, 902 Battelle Boulevard, Richland, WA 99 354, USA.

**Table of Content**

This supplementary information document includes:

Additional experimental section.

Supplementary equations (Equation 1) and calculation.

Supplementary figures (Figure 1S -13S).

Supplementary video (Video S1)

**Additional experimental section**

*Zeta surface potential for blebs.* Measurements (n = 3) were performed on Malvern Zetasizer Nano. A total volume of 1 mL of diluted sample was added in a Zetasizer cuvette for zeta surface potential measurements. 100 µl of bleb sample was mixed with 900 µl of mili-Q water.

*Western blot.* The bleb samples were lysed with Pierce RIPA buffer (Thermo Fisher Scientific, 89900) mixed with 1/200 (v/v) protease inhibitor cocktail (Thermo Fisher Scientific, 1862209) and 1/200 (v/v) EDTA (0.5M) solution (Thermo Fisher Scientific, 1861274) with sample to RIPA buffer ratio 1:1. The bleb lysates were diluted at a 1:1 ratio with 4X Laemmli buffer (Bio-Rad, 1610747) supplemented with β-mercaptoethanol (Bio-Rad, 1610710) and then resolved by SDS-Page Electrophoresis in combination with a ladder (Thermo Fisher Scientific, 26634). Proteins were transferred to a PVDF membrane (Bio-Rad, 1704156) using the Bio-Rad Trans-Blot Turbo Transfer System. The membranes were dried, rewetted in methanol for 30 s, and washed 3 times in TBS-t buffer. The membranes were blocked for 1 hr at RT on a rocker in 5mL of blocking solution (Bio-Rad, 12010020). Primary antibody staining was performed overnight at 4°C on a rocker with a rabbit anti-human ACE-2 antibody (Abcam, ab108252) at 1:500 in the blocking solution. The membranes were washed 3 times in TBS-t buffer and then stained with  the IRDye®800CW goat anti-rabbit secondary antibody (LICOR, 926-32211)  at 3:5,000 in the blocking solution for 30 min at RT. Lastly, the membranes were washed 3 times in TBS-t buffer and imaged using the LICOR Odyssey® M Imaging System. Total protein concentration was measured using a Nanodrop One Microvolume UV-Vis Spectrophotometer (Thermo Fisher Scientific) from 3 technical replicates per sample.

*NTA.* The concentration and size distribution of blebs, VPPs and synthetic liposomes were determined by nanoparticle tracking analysis (NTA, Nanosight NS500, Malvern), with samples diluted in PBS to obtain 20-40 particles/frame. The dilution ratio was multiplied back to obtain the concentration spectra for the final plot.

*PEDOT:PSS and Fabrication of microelectrode array:* The fabricated devices were PEDOT:PSS microelectrode arrays. PEDOT:PSS was used as the interface material combines properties of mechanical compliance and mixed ion/electron conductivities, maintaining biocompatibility relevant for the plasma membrane and higher sensitivity for signal transduction ^[1,2]^. PEDOT:PSS-based electrodes have inherently low impedance (as a result of the volumetric capacitance) compared to inorganic counterparts reduces the baseline signal of frequency-dependent measurements, i.e., Electrochemical impedance spectroscopy (EIS), thereby allows the detection of subtle impedance changes resulting from interactions in biological systems^[3]^.

The microelectrodes were circular electrodes with 450 μm diameters and square electrodes with 200 μm sides. These microelectrode arrays for the experiments discussed in Figure 2-3 are fabricated with parylene C lift-off method. To fabricate the devices, 4-inch glass wafers were first cleaned by sonication in acetone and then isopropanol for 15 minutes. The wafers were rinsed with DI water and baked for 15 min at 150 °C. To pattern for contact tracks, a negative photoresist, AZ nLOF2035 (Microchemicals GmbH) was spun on the glass wafer at 3000 rpm for 45s and exposed with UV light using a mask aligner (Karl Suss MA/BA6). The photoresist was developed in AZ 726 MIF developer (MicroChemicals) developer for 28 s. Ti (5 nm)/Au (100 nm) layer as conductive tracks was deposited by e-beam evaporation on top of the wafer and the Ti-Au metal layer was lifted off by soaking in NI555 (Microchemicals GmbH) overnight. Prior to the deposition of a 2 µm layer (sacrificial layer) of parylene C ((SCS), the wafer was soaked with a 3% A174 (3-(trimethoxysilyl)propyl methacrylate) in ethanol solution (0.1% acetic acid in ethanol) for 60 seconds to promote the parylene C adhesion on the wafer. An anti-adhesive layer of Micro-90 in DI water (2% v/v solution) was spun (1000 rpm for 45 seconds), and then the second layer of a 2 µm parylene C (SCS) was deposited. A layer of positive photoresist AZ 10XT (Microchemicals GmbH) was spun at 3000 rpm for 45s and developed in AZ 726 MIF developer (MicroChemicals) for 6 min to pattern electrode areas or OECT channels. Reactive ion etching (Oxford 80 Plasmalab plus) opened the window for deposition of Clevios PH500 PEDOT:PSS (Heraeus). The PEDOT:PSS solution containing 5 vol% ethylene glycol (Thermo scientific Chemicals, 433810010), 0.26 vol% dodecylbenzenesulfonic acid (DBSA, Thermo Fisher Scientific, 325905000), and 1 vol% (3-glycidyloxypropyl)trimethyloxy-silane (GOPS, Sigma-Aldrich, 440167) was spin-coated at 3000 rpm for 45 s. The sample was baked at 90 °C for 1 min, and the sacrificial layer was peeled off. Finally, the sample was put on a hot plate at 130 °C for 1 h before use. Glass wells (8 mm inner diameter) were glued onto the chip with PDMS (dry overnight), and each microelectrode array is isolated by each glass well.

The microelectrode array for Figure 4 is fabricated with “PEDOT:PSS etching method” with Ge mask. PEDOT:PSS electrodes are patterned on Borofloat 33 glass wafers using standard photolithographic processes in a cleanroom. Wafers are cleaned for 20 min in 90% sulfuric acid/hydrogen peroxide solution at 120°C. Gold lines and contacts (5/50/5 nm of Ti/Au/Ti) are deposited using an electron beam evaporator (AJA International) and patterned with a lift-off process using LOL 2000 and SPR 3612 (DuPont) photoresists. 300 nm SiO_2_ insulation layer is deposited using High-density plasma chemical vapour deposition (HDPCVD) (PlasmaTherm Versaline) and gold contacts are exposed using CHF_3_ in an ICP etcher (PlasmaTherm Versaline) after patterning with SPR 3612 photoresist. PH 1000 PEDOT:PSS (Ossila) is mixed with 5% v/v ethylene glycol and sonicated for 5 min. (3-glycidyloxypropyl)trimethoxysilane (GOPS) is added to solution at 1.5% v/v and then sonicated for 7.5 min with an ice pack placed in the water to prevent heating. Wafers with patterned SiO_2_ are plasma treated at 300 W for 30 sec (March Instruments PX-250 Plasma Asher) and then the prepared PEDOT:PSS solution is spin coated at 2000 RPM. Coated wafers are baked at 130°C for 40 min. 200 nm Ge hard mask is e-beam evaporated onto the PEDOT:PSS and electrodes are patterned using SPR 3612 photoresist. Ge and PEDOT:PSS are etched in the ICP etcher using CF4 and O2 with trace CF4 (10:1), respectively. Wafers are diced, and Ge is removed by soaking in water for 2 days.

*Preparation of liposomes:* Twenty-five mg/mL of 1,2-dioleoylsn-glycero-3-phosphocholine (DOPC, 850375C) and 1,2-dioleoyl-3-trimethylammonium-propane (DOTAP), (Avanti Polar Lipids, 890890C) in chloroform were mixed in the desired 4:1 ratio (v/v) and dried under nitrogen. The lipid mix was dried under vacuum at room temperature for one hour, to evaporate the residual chloroform. The lipids were then resuspended in PBS to a concentration of 4 mg/mL. For POPC liposomes, the same dehydration method can be applied, and the final POPC lipids were resuspended in PBS to 4 mg/mL concentration. The solution was frozen at -20°C for at least 5 hours, and then extruded 20 times through a 100 nm membrane (GE Healthcare).

*Formation of synthetic supported lipid bilayers (SLBs).* 100 μL of liposomes (DOPC/DOTAP 4 mg/mL in PBS) were added on PEDOT:PSS based electrodes after 1 minute of O_2_ plasma at 25W and 0.8 mbar. The liposomes were incubated for 20 min and then rinsed with PBS (3x). A poly(ethylene glycol) solution in PBS (PEG8k, 30% w/v) was then added to the well and incubated for another 20 min. Lastly, the SLBs were washed with PBS 5 times before measurements.

*Studies of two pathways of VPP entry:* After forming either Calu-3 or HEK293-ACE2 SLBs on a microelectrode array, the SLBs were incubated for 30 min in PBS at room temperature. Then, the SLB-baseline EIS measurements were taken before the experiments. In the case of Calu-3, 100 μL of 100 times diluted VPPs (around 10^11^ particle/mL) was first added to wash the SLB surface, and then another 100 μL of VPPs with the same concentration was added and incubated for 15 min. After incubation, the VPP-fused SLB was monitored with EIS.

In the case of HEK-ACE2 SLBs, after the SLB-baseline EIS was measured, 100 μL of 100 times diluted VPPs (around 10^11^ particles/mL) was first added to wash the SLB surface, and another 100 μL of VPPs with the same concentration was added for 15 min incubation. Then, the EIS was taken. 1 μL 0.5 mg/mL cathepsin-L/MEP protein (Abcam, ab81780) was added to the SLB, and the SLB needed to be incubated 15 mins before EIS measurement. After EIS measurement, 100 μL of pH 4.5 PBS (1 mM HCl was titrated into pH 7.4 PBS until pH adjusted to 4.5) buffer (the low pH buffer for triggering fusion) was slowly pipetted into the well to wash the SLB 1 time and another 100 μL pH 4.5 PBS buffer was added for 15 min incubation. After incubation, the VPP-fused SLBs were monitored with EIS.

*References:*

[1] C. Pitsalidis, A.-M. Pappa, A. J. Boys, Y. Fu, C.-M. Moysidou, D. van Niekerk, J. Saez, A. Savva, D. Iandolo, R. M. Owens, *Chem. Rev.* **2021**, acs. chemrev.1c00539.

[2] Z. Lu, A. Pavia, A. Savva, L. Kergoat, R. M. Owens, *Mater. Sci. Eng. R Reports* **2023**, *153*, 100726.

[3] Z. Lu, D. Van Niekerk, A. Savva, K. Kallitsis, Q. Thiburce, A. Salleo, A.-M. Pappa, R. M. Owens, *J. Mater. Chem. C* **2022**, *10*, 8050.

The VPP concentration is calculated by:

$C= \frac{\bar{N}\times(\frac{A_{drop}}{A_{frame}})}{V} \times F$ **Equation (1)**

Where *C* is VPP concentration. $\bar{N}$ is VPP count (number) within the frame of structured illumination microscopy (SIM). $A_{drop}$ is the average area of a drop of VPP sample spread between two cover slip. $A_{frame}$ is the area of frame size of SIM. $V$ is the volume of the VPP sample drop. F is dilution factor.

Here, we calculated the original VPP sample concentration from the 10^-4^ diluted sample ($F$ = 10^4^) since the particles are within a feasible counting range. $\bar{N}$ is calculated by the equation descripting the calibration curve in Figure 7S caption.

When *x* = 10^-4^ is plugged:

$\bar{N}$ = 43.539 VPP

$A_{drop}$ is calculated by analysing the drop spreading images with ImageJ^®^:

$A_{drop}$= 127.816 ± 34.292 mm^2^ = 127,816,000 ± 34,292,000 µm^2^

$A_{frame}$ is calculated by multiplying the SIM frame dimension (55.04 µm for each side):

$A_{frame}=$ 55.04 µm $\times$55.04 µm = 3029.4016 µm^2^

$V$ is known by pipetting volume:

$V=0.5$µL = 5 $\times$ ${10}^{-4}$ mL

Once the numbers are plugged into the Equation 1,

$C$ = $3.67\times$ ${10}^{13}$ VPP/mL

**Figure S1**: the full blot image of the Western blot in Figure 2 (e): Western blot (Biological repeats: n = 2) of Calu-3, HEK293-ACE2, and HEK293 blebs stained with anti-ACE2 antibody. The expected ACE2 protein bands are shown at ~130 kDa. The measured total protein, measured ACE-2 fluorescence, and normalized protein expression are shown matching each lane conditions.


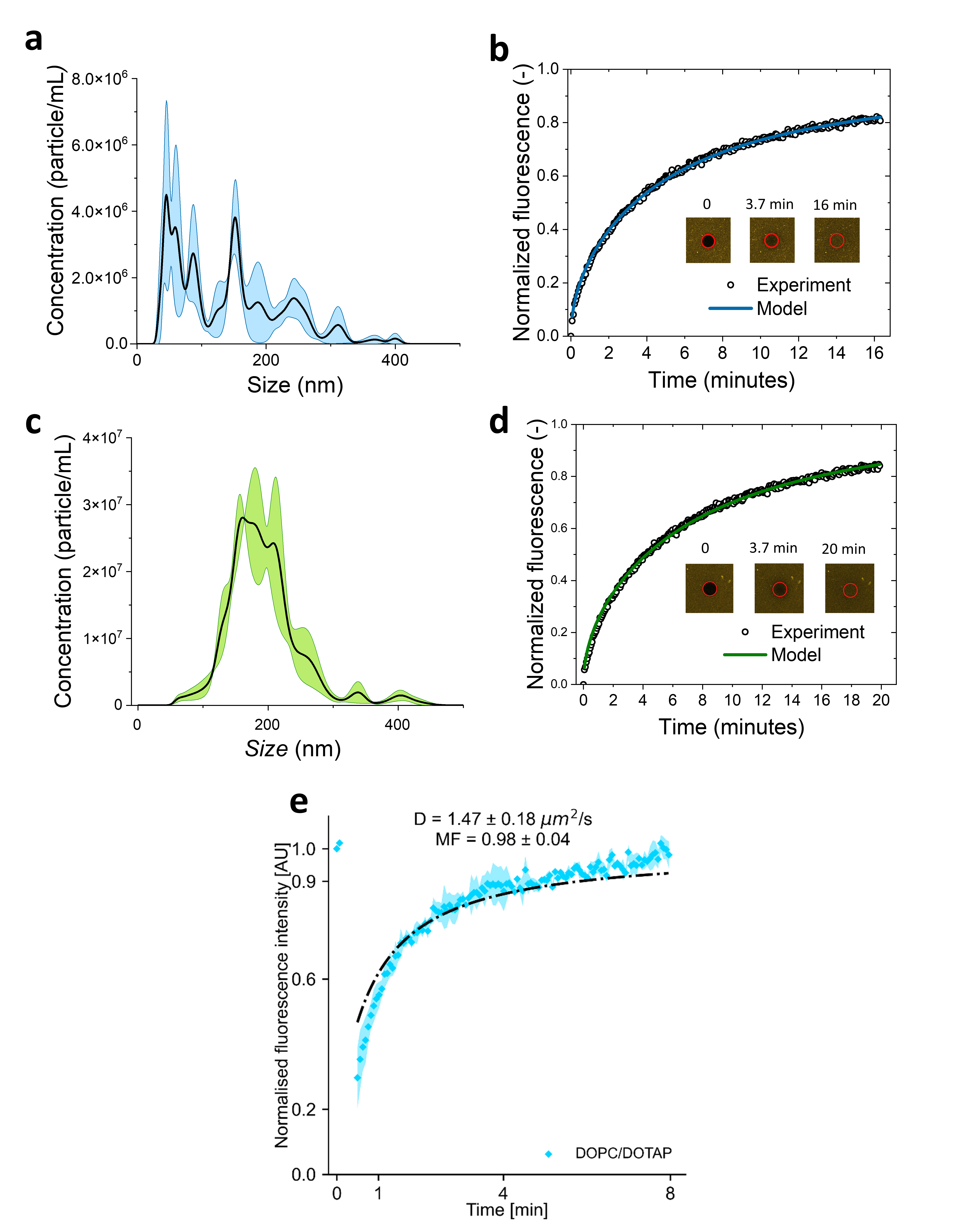


**Figure S2**: Characterization of Calu-3 and HEK293-ACE2 blebs and SLBs: (a), Calu-3 bleb size distribution detected by nanoparticle tracking analysis (NTA) (144.2 ± 3.8 nm is the average size, n = 3). (b), FRAP of Calu-3 SLB with rhodamine B18 (R18) stain formed on PEDOT:PSS coated glass slides. (c), HEK293-ACE2 bleb size distribution detected by NTA (193.5 ± 1.1 nm is the average size n = 3). (d), FRAP of HEK293-ACE2 SLBs with R18 stain formed on PEDOT:PSS coated glass slides. (For (b) and (d), experimental data points and model fitting are presented as circular dots and colored curves, respectively). (e), A typical FRAP measurement on a DOPC:DOTAP SLB formed on PEDOT:PSS thin film coated glass slide (D and MF are reported in the plot).


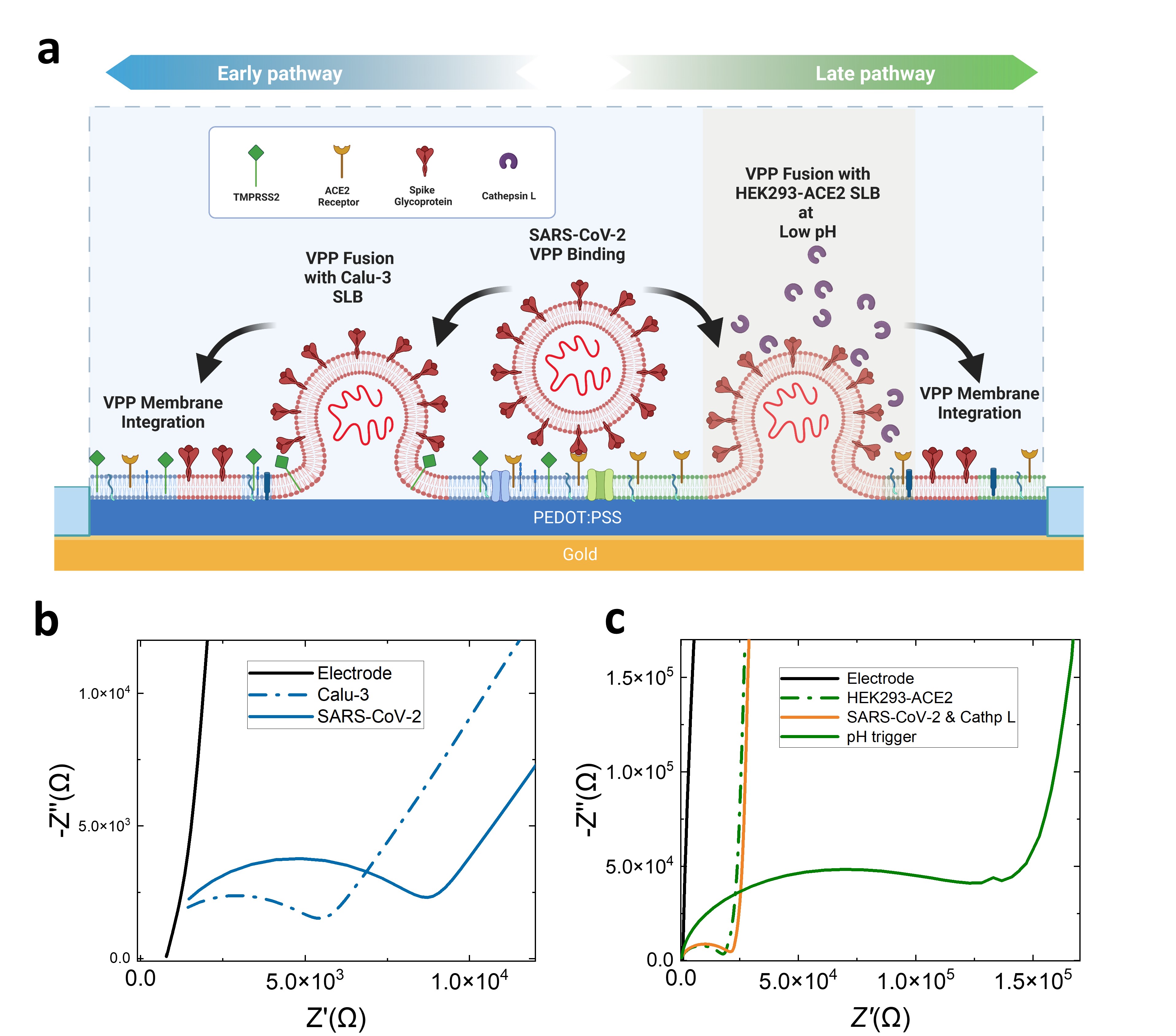


**Figure S3**: (a), Schematic of the early and late pathways of the virus fusion process of SARS-CoV-2 VPPs. (b) – (c) Nyquist plot of representative EIS measurements in Figure 2(h) and (i). (b), fusion on Calu-3; (c), fusion on HEK293-ACE2 with addition of cathepsin L (orange curve) at low pH environment (solid green curve).

**
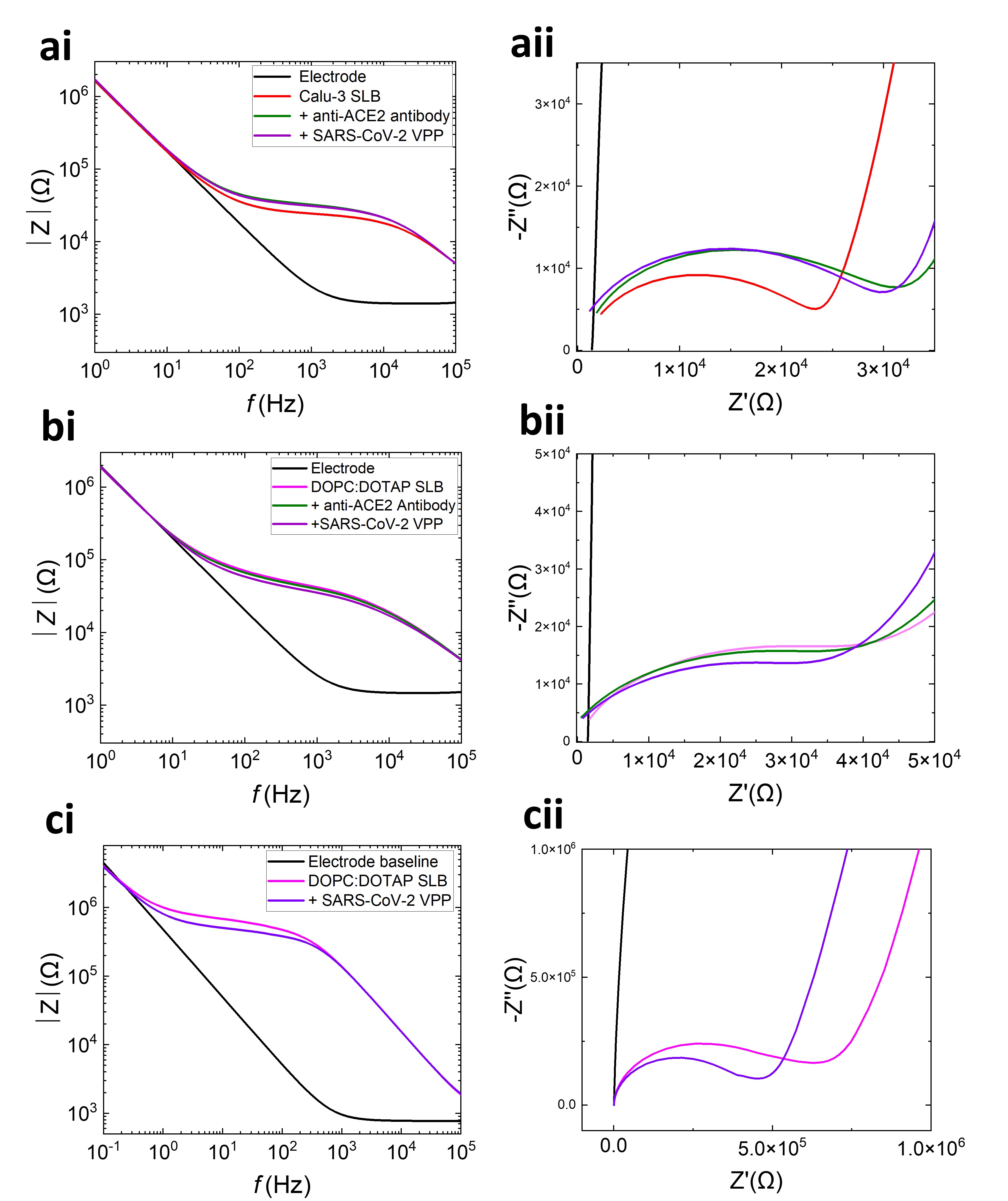
**

**Figure S4**: Electrochemical impedance spectroscopy (EIS) of SARS-CoV-2 VPP inhibition on Calu3 SLBs with anti-ACE2 antibody. (a), VPP fusion on anti-ACE2 antibody treated Calu-3 SLBs ((ai) Bode and (aii) Nyquist plots for (a)). (b), VPP fusion on anti-ACE2 antibody treated DOPC:DOTAP SLBs ((bi) Bode and (bii) Nyquist plots for (b)). (c), VPP fusion on pristine DOPC:DOTAP SLBs ((ci) Bode and (cii) Nyquist plots for (c)). The plots here are the representative EIS spectra for each experiment, from which membrane resistance values were extracted. The summarized resistance changes are presented in Figure 3c. The Z’ and Z’’ refer to the real and imaginary components of the impedance Z, respectively.


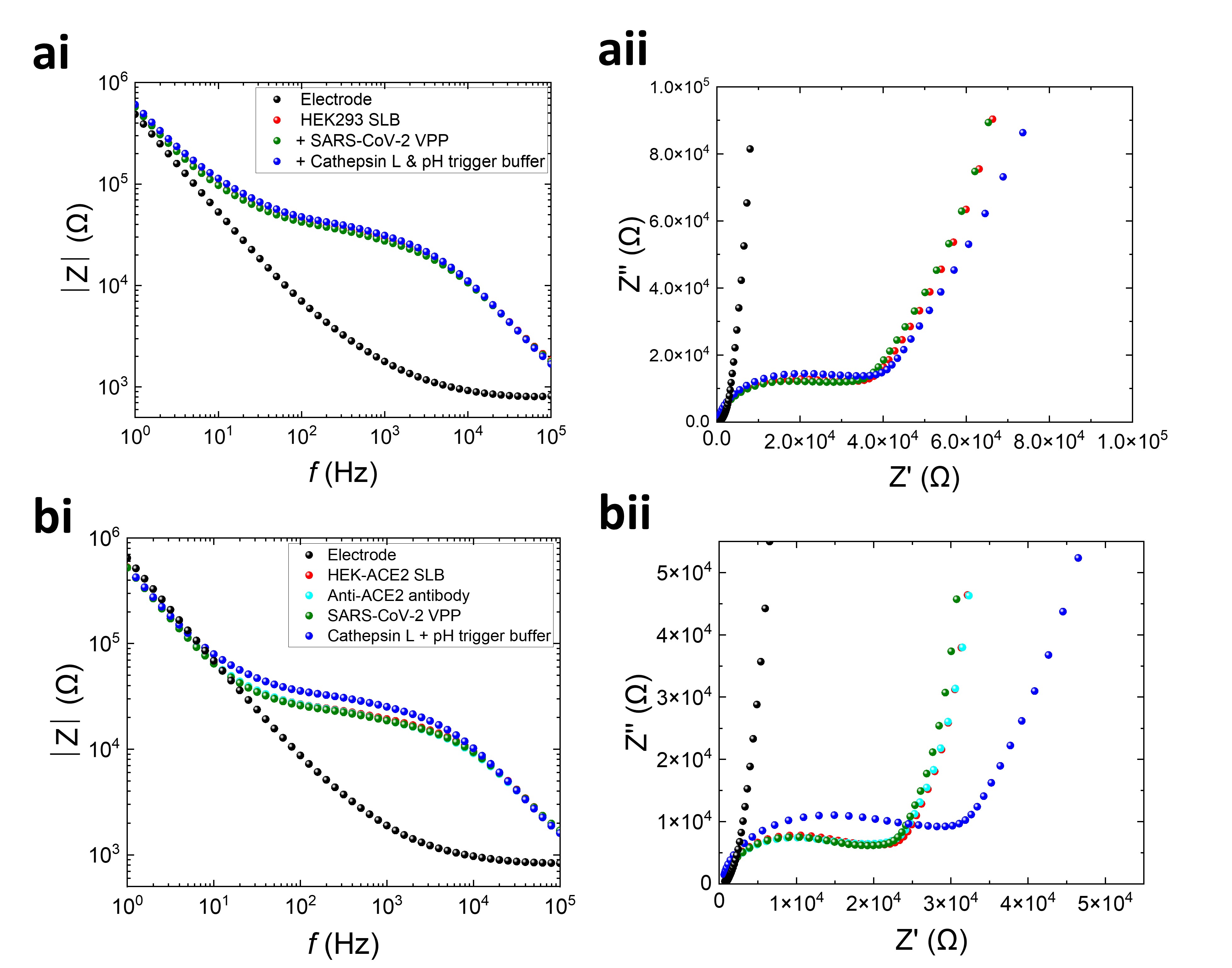


**Figure S5**: EIS measurement of SARS-CoV-2 VPP interactions with HEK293 SLBs and HEK293 overexpressed with ACE2 (HEK293-ACE2) SLBs treated with anti-ACE2 antibody: (a), VPP fusion test on pristine HEK293 SLBs. (b), VPP fusion on anti-ACE2 antibody treated HEK293-ACE2 SLBs. ((i) Bode and (ii) Nyquist plots.) The plots here are the representative EIS spectra for each experiment, from which membrane resistance values were extracted. The summarized resistance changes are presented in Figure 3c.


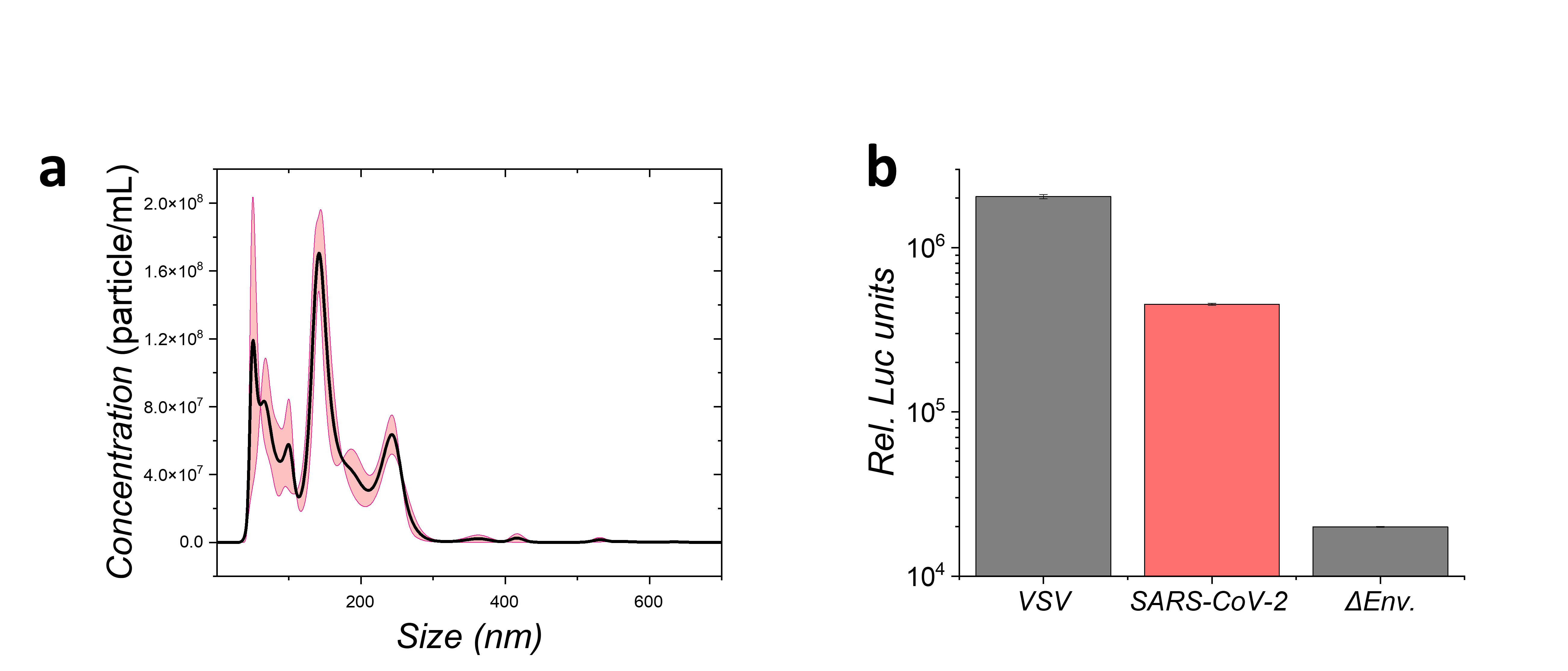


**Figure S6**: (a), Size distribution of SARS-CoV-2 VPPs by NTA with average size (S_VPP,avg_) 151.9 ± 8.2 nm. (b), Luciferase infectivity assay of vesicular stomatitis virus (VSV), SARS-CoV-2 Wuhan-Hu1 particles (SARS-CoV-2), and delta envelope particles (∆Env) on VERO cells. VSV and ∆Env are positive and negative controls, respectively.


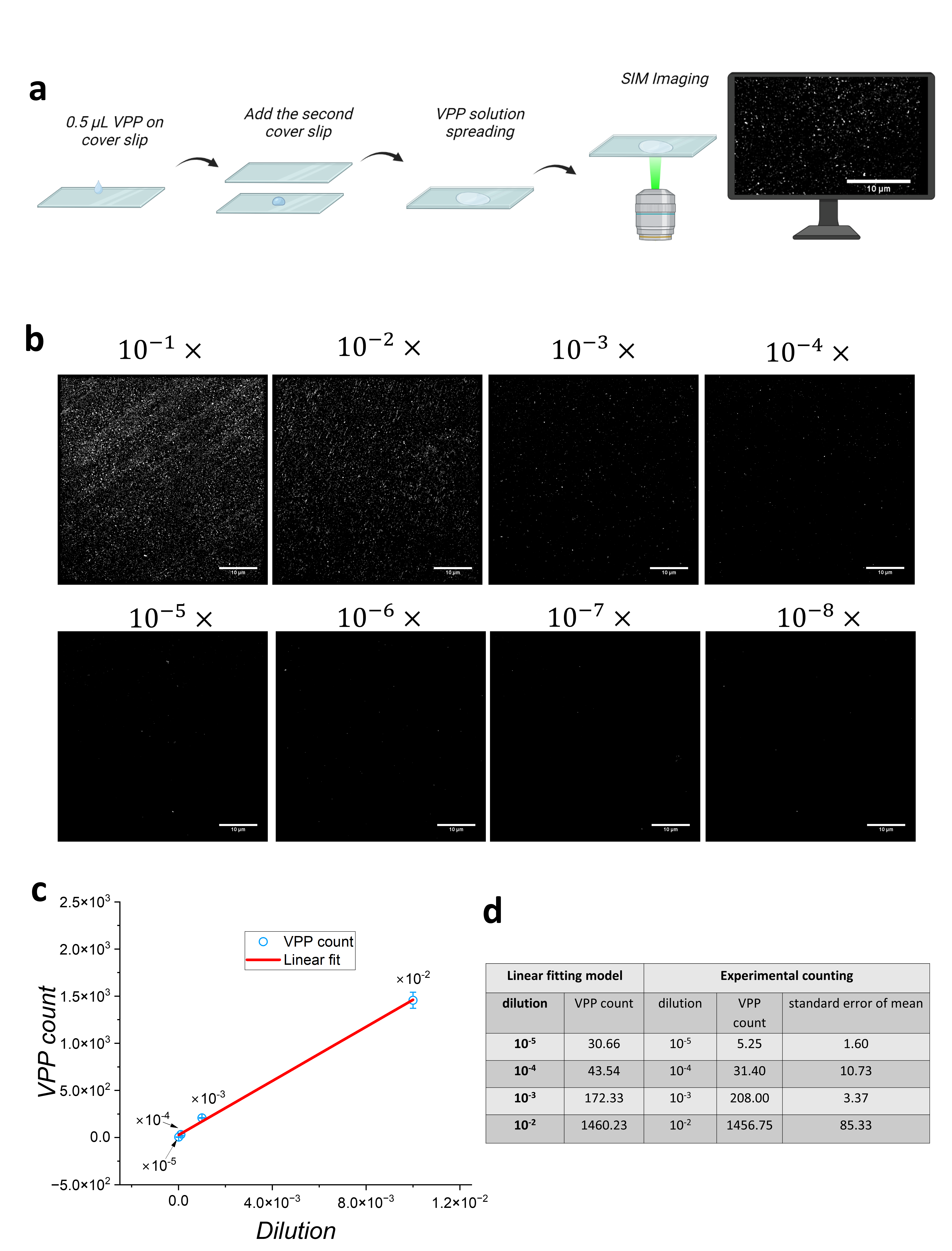


**Figure S7**: Determination SARS-CoV-2 VPPs concentration SIM: (a) Sample preparation steps for SIM imaging (from left to right): 1. Adding 0.5 µl R18-stained VPPs onto one clean cover slip; 2. Adding second cover slip on top; 3. VPP sample spreading between two slides; 4. Imaging with SIM. (b) The representative VPP images at each dilution, scale bar, 10 µm. (c) Calibration curve of VPP count versus dilution (Each dilution is labeled next to each data point. Technique replication with n = 4 for each point for the same batch of VPP in Figure 3), and the red fitting line is described by the equation *y* = **a***x*+**b** (**a** = 143100; **b** = 29.229; R^2^ = 0.9986). (d) Table of comparison of experimental counting values and linear fitting model from (c). When the dilution reaches 10⁻⁵, a significant discrepancy arises between the linear fitting model and the experimental counting model, which may be attributed to systematic dilution errors at low concentrations.


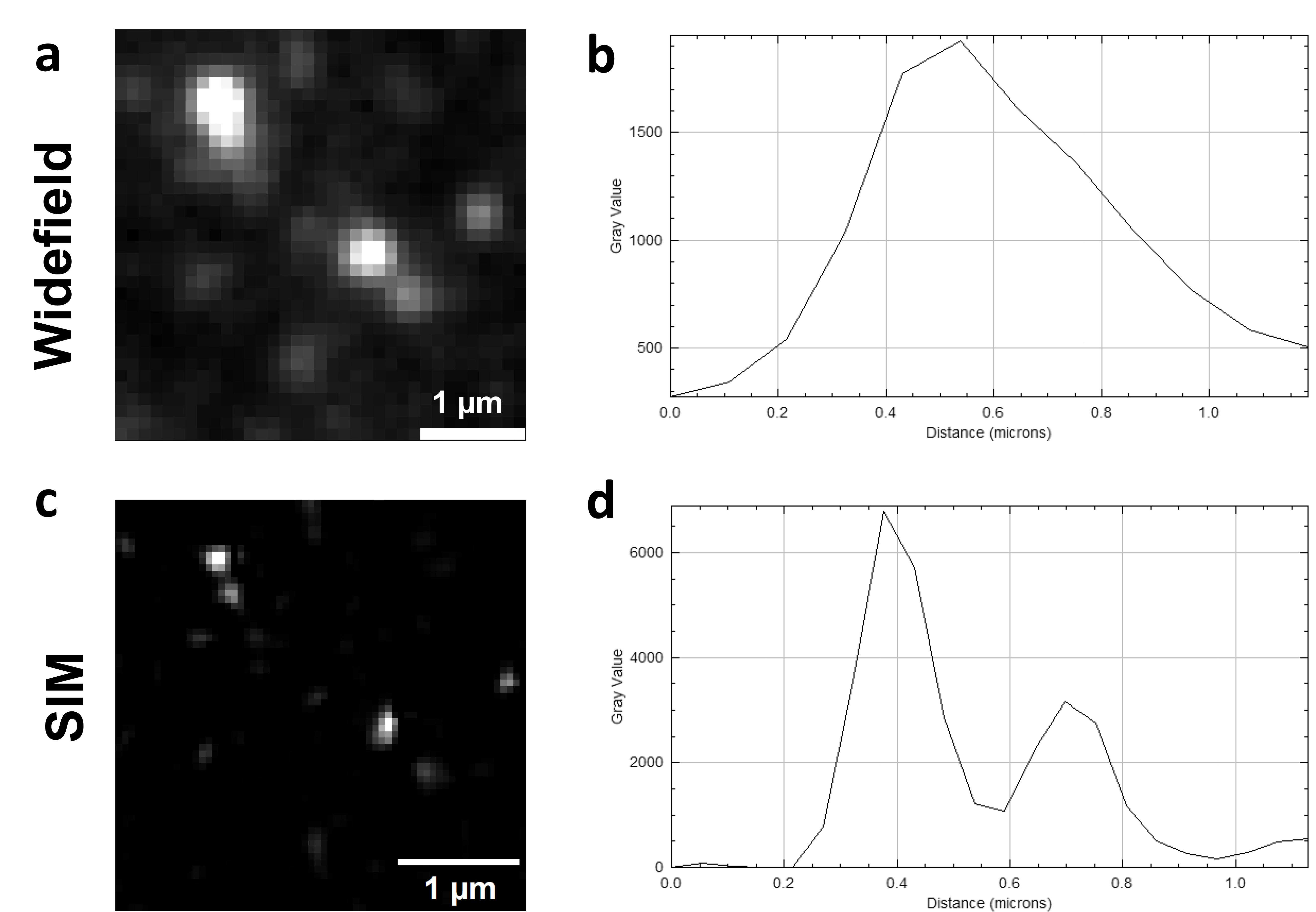


**Figure S8**: The comparison of traditional widefield microscopy and SIM observing on the same sample area: (a), widefield image; (b) the intensity plot of (a). (c), The SIM image; (d), the intensity plot of (c).


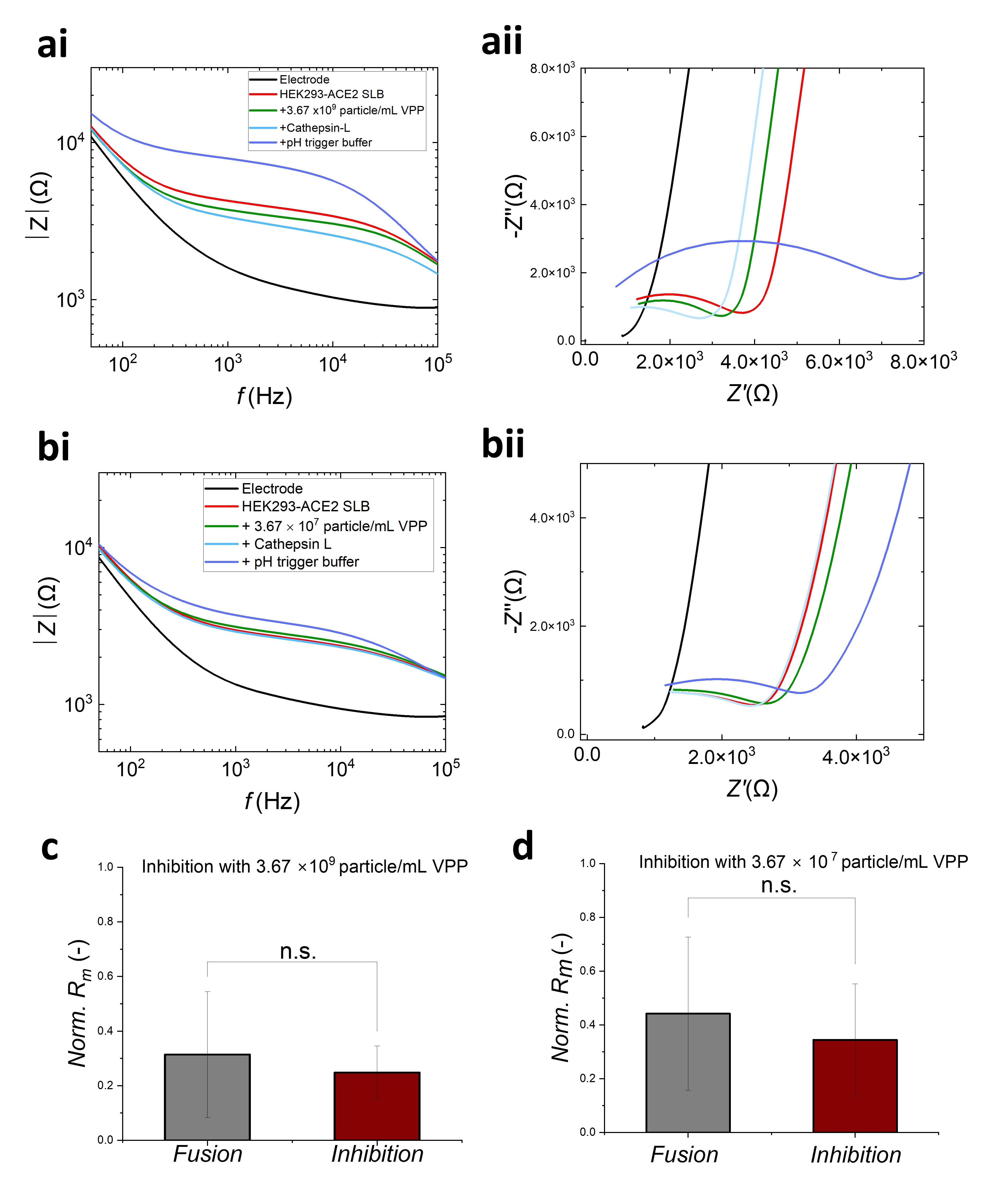


**Figure S9**: Fusion tests of pristine VPP and anti-S treated VPP on HEK293-ACE2 SLB**:** (a), 3.67 x 10^9^ particles/mL VPP fusion test on HEK293-ACE2 SLBs, (b) 3.67 x 10^7^ particle/mL VPP fusion test on HEK293-ACE2 SLBs. The plots here are the representative EIS spectra for each experiment. ((i) Bode and (ii) Nyquist plots.) (c) The comparison of normalized R_m_ increases with 3.67 x 10^9^ particle/mL VPP fusion with (inhibition) and without (fusion) anti-S antibody (10 µg/mL) blocking (n ≥ 3). (d) The comparison of normalized R_m_ increases with 3.67 x 10^7^ particle/mL VPP fusion with (inhibition) and without (fusion) anti-S antibody (10 µg/mL) blocking (n ≥ 3).


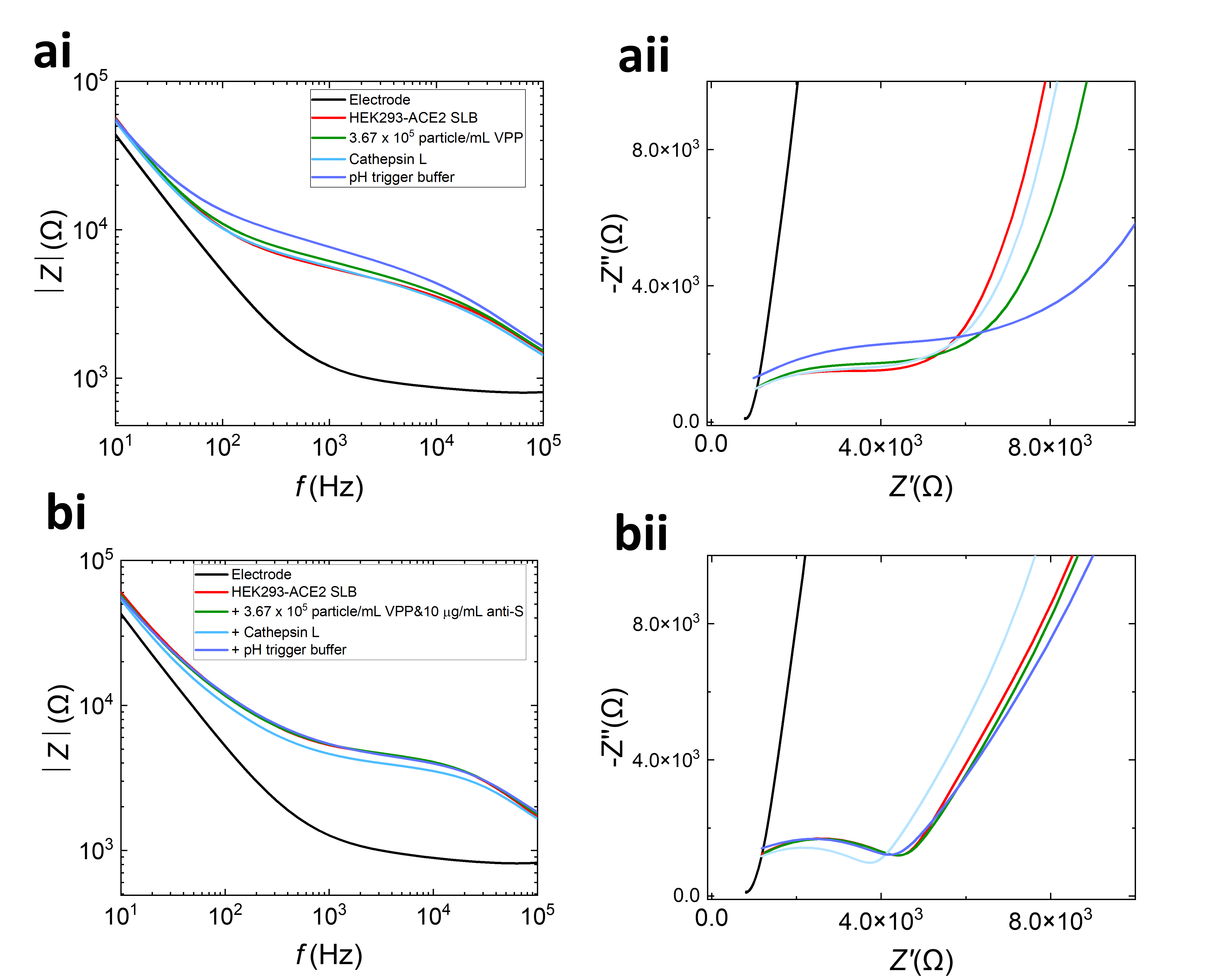


**Figure S10**: Inhibition of SARS-CoV-2 VPP fusion with HEK293-ACE2 SLB: (a), test of 3.67 x 10^5^ particle/mL pristine VPP fused on HEK293-ACE2 SLBs, (b) test of 3.67 x 10^5^ particle/mL VPP fusion incubated with 10 µg/mL anti-S antibody on HEK293-ACEs SLBs. ((i) Bode and (ii) Nyquist plots.)

**Figure S11**: Test of the impact of washing and incubation with PBS on HEK293-ACE2 SLBs: The comparison of R_m_ of HEK293-ACE2 SLBs before (green) and after (grey) washing and incubating with PBS (green). The PBS was added onto HEK293-ACE2 SLBs and pipetted up and down 10 times, and the SLBs were incubated 10 minutes before monitoring with EIS.


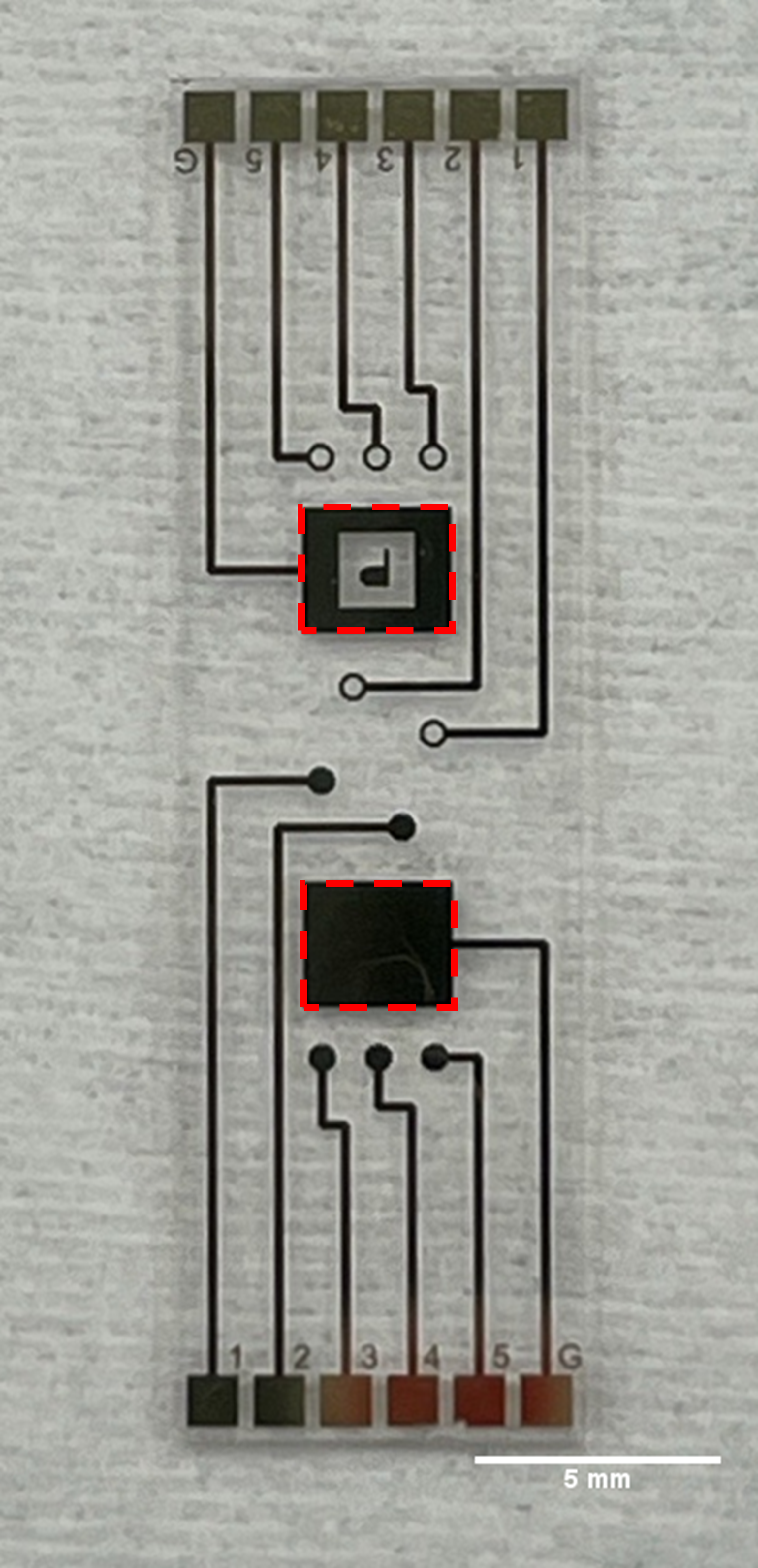


**Figure S12:** The top view of device arrangement: the large PEDOT:PSS counter electrodes are labeled with red dashed box. Each counter electrode has 3mm х 2.5mm rectangular PEDOT:PSS area coated onto gold.


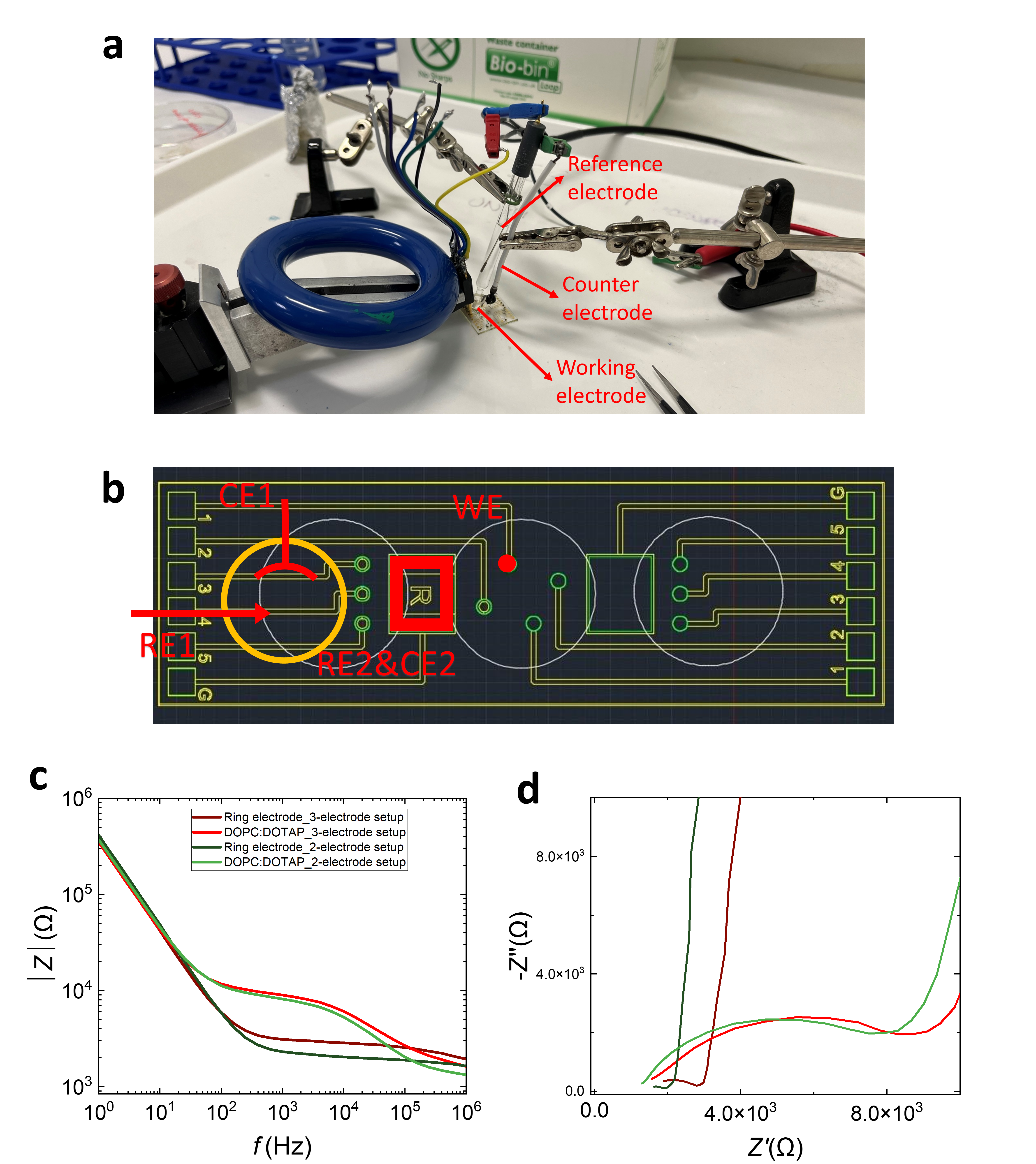


**Figure S13**: EIS comparison between 3-electrode configuration and 2-electrode configuration (co-planar counter electrode): (a) 3-electrode configuration with hanging reference and counter electrodes, which are applied for the devices in Figure 2 and 3. (b) the schematic of electrodes arrangement for measuring the DOPC:DOTAP SLB on working electrode (WE) (ring electrode 1 is labeled with solid red dot): 1^st^ setup is the 3-electrode configuration with the WE, (counter electrode 1) CE1 and (reference electrode 2) RE2; 2^nd^ setup is the 2-electrode configuration with WE and co-planar electrode as the counter electrode (RE2&CE2, where counter and reference electrodes are shorted). (c)-(d) the EIS spectra of the measurements by the two setups (red: 1^st^ setup; green: 2^nd^ setup).
